# Elucidating the biotechnological potential of the genera *Parageobacillus* and* Saccharococcus* through comparative genomic and pan-genome analysis

**DOI:** 10.1186/s12864-024-10635-1

**Published:** 2024-07-25

**Authors:** Michael Mol, Pieter de Maayer

**Affiliations:** School of Molecular & Cell Biology, Faculty of Science, University of the Witwatersrand, Johannesburg, 2000 South Africa

**Keywords:** *Parageobacillus*, *Saccharococcus*, *Geobacillus*, Genomics, Pan-genome, Biotechnology, Thermophile

## Abstract

**Background:**

The genus *Geobacillus* and its associated taxa have been the focal point of numerous thermophilic biotechnological investigations, both at the whole cell and enzyme level. By contrast, comparatively little research has been done on its recently delineated sister genus, *Parageobacillus.* Here we performed pan-genomic analyses on a subset of publicly available *Parageobacillus* and *Saccharococcus* genomes to elucidate their biotechnological potential.

**Results:**

Phylogenomic analysis delineated the compared taxa into two distinct genera, *Parageobacillus* and *Saccharococcus*, with *P. caldoxylosilyticus* isolates clustering with *S. thermophilus* in the latter genus. Both genera present open pan-genomes, with the species *P. toebii* being characterized with the highest novel gene accrual. Diversification of the two genera is driven through the variable presence of plasmids, bacteriophages and transposable elements. Both genera present a range of potentially biotechnologically relevant features, including a source of novel antimicrobials, thermostable enzymes including DNA-active enzymes, carbohydrate active enzymes, proteases, lipases and carboxylesterases. Furthermore, they present a number of metabolic pathways pertinent to degradation of complex hydrocarbons and xenobiotics and for green energy production.

**Conclusions:**

Comparative genomic analyses of *Parageobacillus* and *Saccharococcus* suggest that taxa in both of these genera can serve as a rich source of biotechnologically and industrially relevant secondary metabolites, thermostable enzymes and metabolic pathways that warrant further investigation.

**Supplementary Information:**

The online version contains supplementary material available at 10.1186/s12864-024-10635-1.

## Background

The genus *Geobacillus* has served as an epicentre for biotechnological exploitation of thermophilic taxa [1, 2]. First described following the 16 s rRNA gene-based reclassification of previously recognised thermophilic clustering (group 5) *Bacillus* spp. [3], the genus currently comprises 12 validly described species [4]. Members are Gram-positive, aerobic or facultatively anaerobic, spore forming rods that are characterised by their thermophilicity, being capable of growth at temperatures ranging between 37–80˚C [5]. Key taxa of biotechnological value include *Geobacillus stearothermophilus, G. thermoleovorans* and *G. thermodenitrificans* [2, 6]*.* These and other taxa in the genus have been the topic of research and commercial development in a wide range of whole-cell applications, including bioremediation, crude oil recovery and refinement, textile processing, synthesis of nanoparticles, production of antibiotics and production of value added chemicals such as biodiesel, lactate and ethanol [2, 5, 6]. Geobacilli further serve as a source of various thermostable enzymes which present comparably more cost-effective, rapid, non-toxic and environmentally friendly alternatives to whole-cell or abiotic processes that support diverse industries [5, 6]. The application of thermophile derived enzymes has become more prevalent due to their greater thermostability, pH tolerance, catalytic efficiency and reduced cost and contamination rates associated with thermophilic operation [7]. *Geobacillus-*derived enzymes including α-amylases, α-glucosidases, cellulases, lipases, pectinases, xylanases have received extensive interest for their applicability towards agricultural, biofuel, food, paper, petrochemical, pharmaceutical and textile industries [2, 5, 6].

The application of whole genome phylogenetic approaches highlighted the clustering of *Geobacillus* taxa in two distinct clades, which were further distinguished based on GC content, resulting in the establishment of the genus *Parageobacillus* [8]. This genus currently comprises six validly described species which are readily isolated from diverse and globally distributed high temperature environments including hot springs, oil wells, hot composts and geothermal sites and sediments [5]. Another sister genus of both *Geobacillus* and *Parageobacillus*, *Saccharococcus* was established in 1984 and originally comprised a single species, *S. thermophilus*, isolated from beet sugar extracts [9]. A second thermophilic and xylanolytic species isolated from soil in Australia, *S. caldoxylosilyticus*, was subsequently described [10] but its taxonomic status was short lived, shifting first to the genus *Geobacillus* and subsequently the genus *Parageobacillus* [8].

In congruence with their wide and varied distribution, the genera *Parageobacillus* and *Saccharococcus* encompass a broad range of microorganisms with versatile metabolic potential, encoding a range of robust thermostable and thermoactive enzymes, many of which may be of biotechnological value [2, 5]. While some research has focused on the biotechnological potential of *P. thermoglucosidasius*, the inherent capacity of the genera *Parageobacillus* and *Saccharococcus* as a whole, in comparison to the sister genus *Geobacillus*, remains relatively underexplored. Here we have made use of whole genome sequence data and phylogenomic approaches to establish the relationship of taxa the genera *Parageobacillus* and *Saccharococcus* and demonstrate the clustering of *S. caldoxylosilyticus* with *S. thermophilus* in the latter genus. Further, using comparative genomic and pan-genome analyses, we provide an in depth characterisation of the biotechnological potential of these key thermophilic taxa.

## Results & discussion

### Phylogenomic analysis delineates *Parageobacillus *and *Saccharococcus* as two distinct genera

**Fig. 1:**
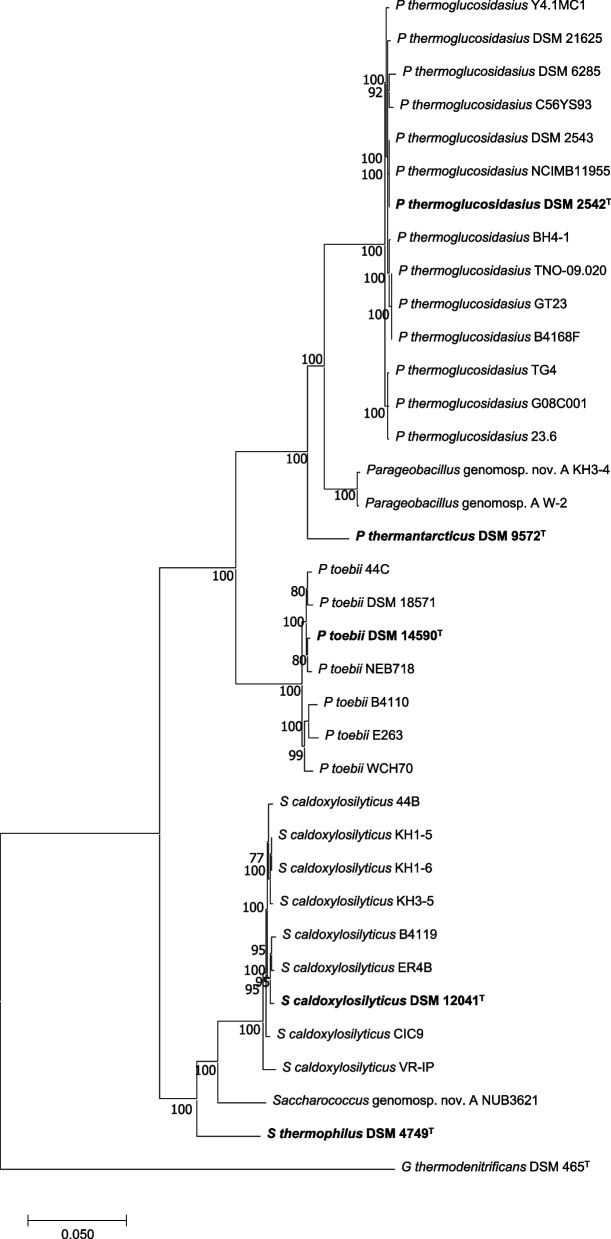
Core genome ML phylogeny of the genera *Parageobacillus* and *Saccharococcus. *The phylogeny was constructed on the basis of 1,784 SCOs, with the concatenated alignment comprising 499,928 amino acid positions, of which 81,368 were deemed parsimony informative and 58,513 represented single sites. The ML phylogeny was constructed using the optimal evolutionary model JTT + F + I + G4 with ultrafast bootstrap support (*n* = 1,000 replicates). *G. thermodenitrificans* DSM 465^T^ was used as outgroup

The genus *Parageobacillus* was resolved from the genus *Geobacillus* using phylogenomic analysis, and comprises six distinct species, including *Parageobacillus caldoxylosilyticus* [8]. However, the taxonomic status of the latter species remains contentious, having first been assigned to the genus *Saccharococcus* [10], subsequently the genus *Geobacillus* and finally the genus *Parageobacillus* [8]. In this study a core genome maximum likelihood phylogeny was constructed on the basis of 1,784 single-copy orthologous proteins conserved among 34 *Parageobacillus* strains, the *Saccharococcus thermophilus* DSM 4749^ T^ genus type and the outgroup strain *Geobacillus thermodenitrificans* DSM 465^ T^. This phylogeny showed the clear delineation of the taxa in two distinct clades (Fig. 1), with the nine *P. caldoxylosilyticus* strains and *Parageobacillus* genomosp. 1 NUB3621 clustering with *S. thermophilus* DSM 4749^ T^, indicating they belong to the genus *Saccharococcus*. This is further supported by the Average Nucleic acid Identity (ANI) and digital DNA-DNA Hybridisation (dDDH) phylogenomic metrics, where intraclade ANI values of 92.22 and 96.16% and dDDH values of 59.19 and 73.72% are observed for the *Parageobacillus* and *Saccharococcus* clades, respectively, while interclade values are 83.57% (ANI) and 27.57% (dDDH) (Additional file 2: Table S1). Two *Parageobacillus* strains with species designation, namely KH3-4 and W-2, demonstrate dDDH (average 44.04%) and ANI (90.01%) values below the 70% and 96% threshold that constitute the species boundaries [8] and as such, they form a novel genomospecies, *Parageobacillus* genomosp. A.

The genomes of members of both *Parageobacillus* and *Saccharococcus* are similar in size (average: 3.763 and 3.742 Mb, respectively), while the genomic G + C contents of members of the genus *Saccharococcus* are on average 0.94% greater than their *Parageobacillus* counterparts (Table 1). The genomes of taxa in both genera code for a similar number of proteins (3,704 and 3,719, respectively), with the most proteins encoded on the genome of *S. caldoxylosilyticus* B4119 (3,986), followed by three *P. thermoglucosidasius* strains. In general, less proteins are encoded on the genomes of *P. toebii* strains (average 3,461 proteins). The least proteins (3,085) are encoded on the genome of *S. thermophilus* DSM 4749^ T^, with a genome that is also ~ 650 kb smaller than the other comparator taxa on average. Analysis of the COG functions associated with the proteomes of each strain showed that slightly more proteins (~ 2% or 84 proteins on average) involved in metabolism are encoded on the genomes of *Saccharococcus* taxa, which can primarily be attributed to the COG categories amino acid (E), nucleotide (F) and lipid (I) transport and metabolism (Additional file 1: Figure S1). By contrast, the outgroup taxon *G. thermodenitrificans* DSM 465^ T^ codes for substantially fewer (2.5%) proteins involved in information processing and storage (primarily in COG category L—replication, recombination and repair) and a greater (3.2%) number of proteins of unknown function than the two comparator genera (Additional file 1: Figure S1).

**Table 1:** Metadata of the taxa and genomes used for comparative genomic and phylogenomic analysis

**Organism Name**	**Strain**	**Assembly Accession**	**Bioporject**	**Biosample**	**Assembly Level**	**WGS project accession**	**Isolation source**	**Country**	**BUSCO completeness (%)**	**# contigs/replicons**
*Parageobacillus thermantarcticus*	DSM 9572^T^	GCA_900111865.1	PRJEB17059	SAMN05192569	Scaffold	FOJS01	Geothermal soil	Antarctica	99.1	9
*Parageobacillus thermoglucosidasius*	23.6	GCA_024509915.1	PRJNA668107	SAMN16397577	Complete Genome	CP063414.1-CP063417.1	Soil	Spain	99.1	3
*Parageobacillus thermoglucosidasius*	B4168	GCA_001587555.1	PRJNA270597	SAMN03267297	Contig	LQYU01	Dairy product	Netherlands	99.1	1
*Parageobacillus thermoglucosidasius*	BH4-1	GCA_022846475.1	PRJDB12551	SAMD00442892	Complete Genome	AP025621.1-AP025622.1	Non-contaminated soil	Japan	99.1	2
*Parageobacillus thermoglucosidasius*	C56-YS93	GCA_000178395.2	PRJNA40781	SAMN02232024	Complete Genome	CP002835.1-CP002837.1	Hot Spring	USA	99.1	3
*Parageobacillus thermoglucosidasius*	DSM 2542^T^	GCA_001295365.1	PRJNA296418	SAMN04099008	Complete Genome	CP012712.1	Soil	Japan	98.6	3
*Parageobacillus thermoglucosidasius*	DSM 2543	GCA_014218625.1	PRJNA482718	SAMN09711376	Contig	QQOJ01	Soil	Japan	99.1	5
*Parageobacillus thermoglucosidasius*	DSM 6285	GCA_014218645.1	PRJNA482719	SAMN09711377	Contig	QQOK01	River sediment	USA	99.1	9
*Parageobacillus thermoglucosidasius*	DSM 21625	GCA_014218665.1	PRJNA482720	SAMN09711378	Contig	QQOL01	Flax plants	Germany	99.1	22
*Parageobacillus thermoglucosidasius*	G08C001	SRX1619421	PRJNA311332	SAMN04549607	Contig	SRX1619421	Soil	USA	99.1	14
*Parageobacillus thermoglucosidasius*	GT23	GCA_001651535.1	PRJNA314192	SAMN04532072	Scaffold	LUCT01	Casein pipeline	Netherlands	99.1	1
*Parageobacillus thermoglucosidasius*	NCIMB 11955	GCA_001700985.1	PRJNA330787	SAMN05416582	Complete Genome	CP016622.1-CP016624.1	TMO Renewables	United Kingdom	98.9	3
*Parageobacillus thermoglucosidasius*	TG4	GCA_003865195.2	PRJDB7652	SAMD00150856	Scaffold	BHZK01	Marine sediment	Japan	99.1	3
*Parageobacillus thermoglucosidasius*	TNO-09.020	GCA_000258725.1	PRJNA81577	SAMN02471215	Chromosome	AJJN01	Dairy factory biofilm	Netherlands	99.1	1
*Parageobacillus thermoglucosidasius*	Y4.1MC1	GCA_000166075.1	PRJNA33183	SAMN00002562	Complete Genome	CP002293.1-CP002294.1	Hot Spring	USA	99.3	2
*Parageobacillus toebii*	44C	GCA_014679925.1	PRJNA354604	SAMN06347029	Complete Genome	CP061475.1	Gold mine shaft	USA	99.1	2
*Parageobacillus toebii*	B4110	GCA_001587455.1	PRJNA270597	SAMN03267295	Scaffold	LQYW01	Pea soup	Netherlands	99.5	9
*Parageobacillus toebii*	DSM 14590^T^	GCA_003688615.2	PRJNA455457	SAMN09062732	Complete Genome	CP049703.1-CP049704.1	Hay compost	Korea	98	2
*Parageobacillus toebii*	DSM 18751	GCA_002217735.1	PRJNA383662	SAMN06770004	Contig	NDYL01	Compost	Italy	99.6	4
*Parageobacillus toebii*	E263	GCA_007197795.1	PRJNA553850	SAMN12252226	Complete Genome	CP041632.1	Deep-sea hydrothermal vent	China	99.3	1
*Parageobacillus toebii*	NEB718	GCA_016939435.1	PRJNA622823	SAMN17843211	Complete Genome	CP070511.1-CP070513.1	-	-	99.1	3
*Parageobacillus toebii*	WCH70	GCA_000023385.1	SAMN00000635	SAMN00000635	Complete Genome	CP001638.1-CP001640.1	Compost	USA	99.8	3
*Parageobacillus* genomosp. nov. A	W-2	GCA_001655645.1	PRJNA320062	SAMN04915186	Contig	LXMA01	Oil reservoir	China	99.8	11
*Parageobacillus* genomosp. nov. A	KH3-4	GCA_022846435.1	PRJDB12551	SAMD00442693	Complete Genome	AP025627.1	Non-contaminated soil	Japan	99.8	1
*Saccharococcus caldoxylosilyticus*	44B	GCA_014680125.1	PRJNA662697	SAMN06347028	Complete Genome	CP061476.1	Gold mine shaft	USA	99.4	1
*Saccharococcus caldoxylosilyticus*	B4119	GCA_001587505.1	PRJNA270597	SAMN03267290	Scaffold	LQYS01	Dairy product	Netherlands	100	18
*Saccharococcus caldoxylosilyticus*	CIC9	GCA_000313345.1	PRJNA175758	SAMN02470176	Contig	AMRO01	Hot Spring	Indonesia	100	4
*Saccharococcus caldoxylosilyticus*	DSM 12041^T^	GCA_014196025.1	PRJNA583512	SAMN13173495	Scaffold	JACICX01	Soil	Australia	99.3	22
*Saccharococcus caldoxylosilyticus*	ER4B	GCA_019272935.1	PRJNA344835	SAMN06209283	Complete Genome	CP040553.1-CP040554.1	Compost	Malaysia	99.8	2
*Saccharococcus caldoxylosilyticus*	KH1-5	GCA_022846395.1	PRJDB12551	SAMD00442691	Complete Genome	AP025623.1	Non-contaminated soil	Japan	100	1
*Saccharococcus caldoxylosilyticus*	KH1-6	GCA_022846415.1	PRJDB12551	SAMD00442692	Complete Genome	AP025624.1	Non-contaminated soil	Japan	100	1
*Saccharococcus caldoxylosilyticus*	KH3-5	GCA_022846455.1	PRJDB12551	SAMD00442694	Complete Genome	AP025625.1-AP025626.1	Non-contaminated soil	Japan	100	2
*Saccharococcus caldoxylosilyticus*	VR-IP	GCA_013357975.1	PRJNA637488	SAMN15143704	Scaffold	JABVYQ01	Iron particles from steam vent	India	99.8	2
*Saccharococcus* genomosp. nov. A	NUB3621	GCA_000632515.1	PRJNA189971	SAMN02727286	Chromosome	AOTZ01	Soil	China	99.8	6
*Saccharococcus thermophilus*	DSM 4749^T^	GCA_011761475.1	PRJNA332068	SAMN05444710	Contig	JAASRS01	Sugar beet extraction	Sweden	99.6	3
*Geobacillus thermodenitrificans*	DSM 465^T^	GCA_002072065.1	PRJNA347632	SAMN05894115	Complete Genome	CP017694.1	Sugar beet juice	Austria	99.3	1

**Organism Name**	**Genome size (Mb)**	**G+C content (%)**	**# proteins**	**Plasmids**	**% genome/plasmid**	**%proteins/plasmid**	**Phage elements (intact/incomplete)**	**% phages/genome**	**% phage proteins/genome**
*Parageobacillus thermantarcticus*	3.445	43.67	3497	1	1.67	0.86	6 (2/4)	4.19	6.41
*Parageobacillus thermoglucosidasius*	4.024	43.69	3944	2	3.81	4.31	4 (1/3)	2.75	3.35
*Parageobacillus thermoglucosidasius*	3.737	43.85	3686	-	0.00	0.00	1 (1/0)	1.91	1.79
*Parageobacillus thermoglucosidasius*	3.892	43.88	3762	1	2.40	2.50	1 (0/1)	0.57	0.56
*Parageobacillus thermoglucosidasius*	3.994	43.93	3944	2	2.52	2.41	6 (1/5)	2.36	3.22
*Parageobacillus thermoglucosidasius*	3.980	43.84	3863	2	3.07	3.44	3 (1/2)	2.30	3.55
*Parageobacillus thermoglucosidasius*	3.962	43.80	3863	2	3.20	3.47	6 (0/6)	1.38	1.68
*Parageobacillus thermoglucosidasius*	3.968	43.58	3868	1	1.77	1.84	7 (0/7)	1.58	1.99
*Parageobacillus thermoglucosidasius*	4.006	43.83	3946	2	2.32	2.20	1 (0/1)	0.28	0.41
*Parageobacillus thermoglucosidasius*	3.858	43.83	3779	2	2.69	2.75	2 (0/2)	1.20	1.35
*Parageobacillus thermoglucosidasius*	3.694	43.80	3661	-	0.00	0.00	1 (1/0)	1.93	1.80
*Parageobacillus thermoglucosidasius*	3.989	43.83	3871	2	3.30	3.54	6 (0/6)	1.37	1.68
*Parageobacillus thermoglucosidasius*	3.945	43.93	3878	1	2.40	2.37	2 (1/1)	1.71	2.27
*Parageobacillus thermoglucosidasius*	3.773	43.96	3701	-	0.00	0.00	1 (1/0)	1.82	1.70
*Parageobacillus thermoglucosidasius*	3.912	44.01	3817	1	1.83	1.86	2 (1/1)	1.96	2.65
*Parageobacillus toebii*	3.336	42.53	3308	1	1.43	1.42	3 (1/2)	1.81	2.09
*Parageobacillus toebii*	3.526	42.16	3548	1	1.68	1.66	3 (1/2)	2.33	2.54
*Parageobacillus toebii*	3.323	42.36	3281	1	1.59	1.92	2 (0/2)	0.80	0.73
*Parageobacillus toebii*	3.796	41.58	3816	-	0.00	0.00	7 (0/7)	2.76	3.38
*Parageobacillus toebii*	3.478	42.61	3397	-	0.00	0.00	4 (0/4)	1.57	1.41
*Parageobacillus toebii*	3.456	42.22	3441	2	1.56	1.71	3 (0/3)	1.87	2.18
*Parageobacillus toebii*	3.509	42.80	3433	2	1.26	1.54	4 (0/4)	1.02	0.87
*Parageobacillus* genomosp. nov. A	3.899	43.15	3882	1	2.03	1.78	8 (3/5)	5.79	6.29
*Parageobacillus* genomosp. nov. A	3.817	43.41	3725	-	0.00	0.00	4 (0/4)	2.39	2.74
*Saccharococcus caldoxylosilyticus*	3.774	44.27	3698	-	0.00	0.00	4 (0/4)	0.73	0.81
*Saccharococcus caldoxylosilyticus*	3.943	44.02	3986	3	3.23	3.31	4 (2/2)	2.80	3.49
*Saccharococcus caldoxylosilyticus*	3.824	44.18	3757	-	0.00	0.00	2 (1/1)	1.84	2.24
*Saccharococcus caldoxylosilyticus*	3.854	44.12	3764	1	1.50	1.86	2 (0/2)	1.27	0.74
*Saccharococcus caldoxylosilyticus*	3.912	44.32	3863	1	1.39	1.50	2 (1/1)	1.89	2.43
*Saccharococcus caldoxylosilyticus*	3.851	44.32	3783	-	0.00	0.00	3 (0/3)	1.10	0.90
*Saccharococcus caldoxylosilyticus*	3.851	44.32	3782	-	0.00	0.00	3 (0/3)	1.10	0.90
*Saccharococcus caldoxylosilyticus*	3.839	44.19	3753	1	0.18	0.27	1 (0/1)	0.31	0.27
*Saccharococcus caldoxylosilyticus*	3.823	44.04	3809	1	1.15	1.50	4 (1/3)	4.28	4.54
*Saccharococcus* genomosp. nov. A	3.626	44.40	3637	-	0.00	0.00	1 (0/1)	0.93	0.97
*Saccharococcus thermophilus*	3.135	44.90	3085	2	2.60	2.63	3 (1/2)	3.29	4.51
*Geobacillus thermodenitrificans*	3.473	49.13	3384	-	0.00	0.00	2 (2/0)	2.29	3.34

### *Parageobacillus* and *Saccharococcus* have open pan-genomes with *P. toebii* as a key driver of novel gene accrual

**Fig. 2:**
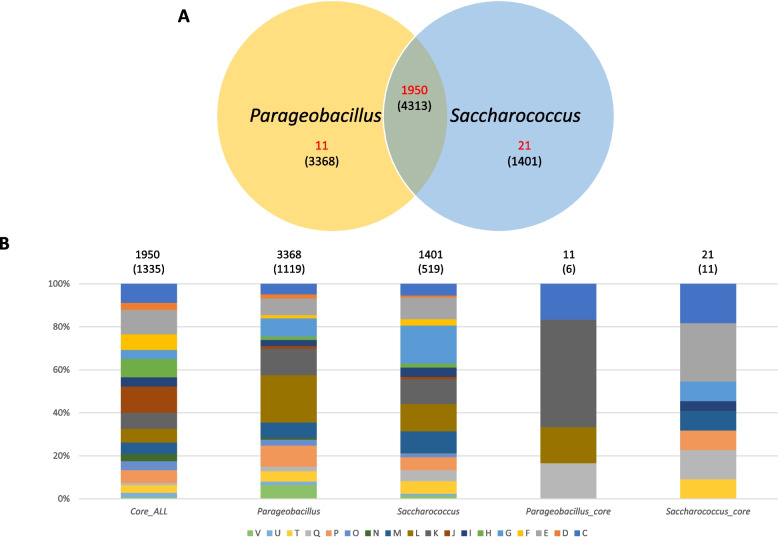
Protein conservation and function in the genera *Parageobacillus* and *Saccharococcus*. **A** Proportion of conserved and genus-specific orthogroups for the two genera. Numbers in red indicate those proteins conserved in all taxa within each dataset, while those in brackets reflect those that are present in some, but not necessarily all taxa within each genus or the combination of the genera. **B** Conserved Orthologous Group functional categories for the proteins conserved among all taxa in both genera, those specific to each genus and those that are conserved among all taxa in each genus. The graphs represent the relative proportions as calculated for those proteins categorized for a COG function (with the exception of category S: function unknown). The number of proteins assigned to COG functional categories are shown in brackets above each bar

The core (conserved among all taxa in a set), accessory (conserved among some taxa or unique to specific taxon in set) and pan-genome (combination of core and accessory fractions) for the genera *Parageobacillus* and *Saccharococcus* were determined. The overall pan-genome of both genera combined (taxa) comprises 9,082 orthogroups, of which 1,950 (21.5%) are core to all taxa (Fig. 2A). A total 37.1% and 15.4% of the orthogroups are unique to the genera *Parageobacillus* and *Saccharococcus*, respectively. Analysis of the functions of the core and *Parageobacillus-* and *Saccharococcus*-unique fractions showed that carbohydrate transport and metabolism (COG category G), in particular, is overrepresented in the genus-specific proteome datasets, suggesting distinct metabolic capacities for the two genera. Furthermore, the synthesis of secondary metabolite biosynthesis (COG category Q) and defense mechanisms (COG category V) are largely genus-specific traits (Fig. 2B). Only eleven and twenty-one orthogroups are core to all *Parageobacillus* and *Saccharococcus* taxa in each set, respectively. The *Parageobacillus*-unique core proteins are dominated by transcription regulators (four proteins), while the *Saccharococcus*-unique core proteins include three proteins involved in amino acid transport and three proteins involved in copper resistance (CotA, CopC and YcnI) (Fig. 2B).

**Fig. 3:**
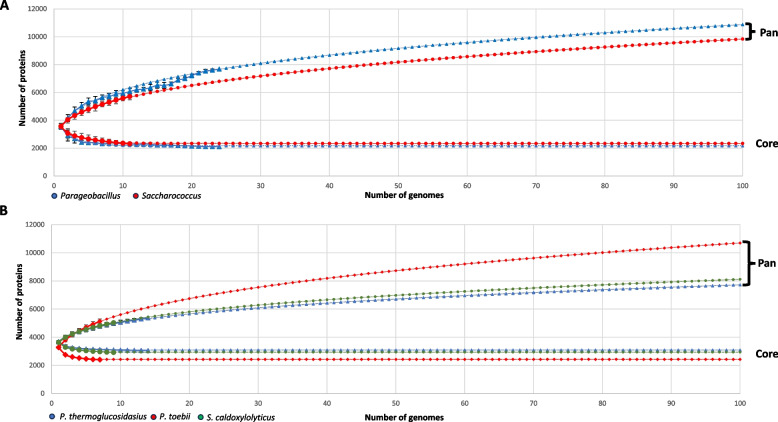
Pan- and core-genome graphs. **A** Pan- and core genomes for the genera *Parageobacillus* (blue) and *Saccharococcus* (red). Larger shapes indicate the actual values, while smaller shapes depict the extrapolated values. **B** The pan- and core genomes of *P. thermoglucosidasius* (blue)*, P. toebii* (red) and *S. caldoxylosilyticus* (green)

Pan- and core genome graphs were constructed for the genera *Parageobacillus* and *Saccharococcus* and extrapolated to encompass 100 genomes/genus (Fig. 3A). Both genera display an open pan-genome, with that of *Parageobacillus* being slightly larger than the genus *Saccharococcus*. Similar numbers of new genes (24.4 and 24.8) are predicted to be added to the pan-genome when the 100th genome of *Parageobacillus* and *Saccharococcus* is sequenced. When considering genome conservation, the core genome of *Saccharococcus* is predicted to be slightly larger (2,332) than that of *Parageobacillus* (2,171) across 100 genomes.

To evaluate the pan-genome dynamics of individual species within each genus, the pan- and core-genomes of three species for which ≥ 7 genomes are available (*P. thermoglucosidasius*—14 genomes*, P. toebii*—7 genomes and *S. caldoxylosilyticus—*8 genomes), were extrapolated (Fig. 3B). All three species display open pan-genomes. Similar pan- and core-genome trends were observed for *P. thermoglucosidasius* and *S. caldoxylosilyticus,* with the core genome approaching a predicted average of 3,012 orthogroups when 100 genomes are sequenced, while the 100th genome would add 15 novel proteins to the pan-genome of both species. By contrast, a much larger pan-genome (~ 2,800 more orthogroups when considering 100 genomes) was observed for *P. toebii* than the other species, with 24.4 novel proteins added by the 100th taxon genome included in the analysis. This species further has a substantially smaller core genome, with almost 600 core orthogroups less than the other two species. This suggests that *P. toebii* has a more unstable pan-genome than *P. thermoglucosidasius* and *S. caldoxylosilyticus* and that this species may be capable of greater ecological, metabolic and functional diversification than the two latter species [11]. This is further supported when considering the genomes incorporated in this study, where *P. toebii-*specific orthogroups (seven genomes) contribute 14.6%, while *P. thermoglucosidasius*-specific proteins (with double the number of genomes analysed) contribute 18.5% (Additional file 1: Figure S2A). The largest proportion of proteins involved in the supra-functional category information storage and processing (38.4%) is observed for the *P. toebii*-specific protein complement, while the *P. thermoglucosidasius*-specific proteins are primarily involved in metabolism (Additional file 1: Figure S2B). These species-specific datasets are predominated by proteins involved in DNA replication, recombination and repair (25.9%) and carbohydrate (10.4%) as well as amino acid (10.1%) transport and metabolism, respectively (Additional file 1: Figure S2C).

### Plasmids, bacteriophages and transposable elements are key drivers of *Parageobacillus *and *Saccharococcus* diversification

Plasmid replicons, prophages and transposable elements were predicted for the comparator *Parageobacillus* and *Saccharococcus* taxa. Plasmid replicons occur in 75% and 54.5% of the taxa in each genus, respectively (Table 1). Half of the plasmid-bearing *Parageobacillus* taxa incorporate two plasmids, while *S. thermophilus* DSM 4749^ T^ harbours two plasmids and *S. caldoxylosilyticus* B4119 is predicted to carry three distinct plasmids. The plasmids vary substantially in size, with the smallest (1,080 nucleotides) and largest (~ 105 kilobases) both occurring in *P. thermoglucosidasius* G08C001. These plasmids contribute up to 3.81% and 4.31% of the total genome and protein complement (highest for both observed in *P. thermoglucosidasius* 23.6) (Table 1). Prophage elements are more prevalent in both genera, with between one and eight (*Parageobacillus* genomosp. A. W-2) elements per genome (Table 1). In most cases these prophage elements are predicted to be incomplete, but three complete phage elements are predicted on the genome of *Parageobacillus* genomosp. A W-2 and phage-proteins contribute 6.29% of the total proteins encoded on the genome of the latter strain.

Between 29 (*Saccharococcus* genomosp. A NUB3621) and 263 (*P. toebii* WCH70) transposases (belonging to 74 distinct orthogroups) were predicted per genome. Notably, *P. toebii* incorporate an average of 124 transposases per genome, while *P. thermoglucosidasius* and *S. caldoxylosilyticus* genomes incorporate an average 50 and 63 transposases, respectively, indicating a key role for transposition in the diversification of *P. toebii*.

When considering plasmids, prophages and transposases in combination, these elements contribute 6.1% and 5.1% of the total genomic protein contents for *Parageobacillus* and *Saccharococcus*, respectively, while for the comparator *G. thermodenitrificans* DSM 465^ T^, these elements encompass only 3.8% of the total proteome. Stand-out taxa include *P. thermantarcticus* DSM 9572^ T^ and *P. toebii* WCH70, where these elements in combination, contribute 9.7% (primarily prophage elements) and 10.1% (primarily transposases) of the total protein content, highlighting the combined role of these elements in shaping the highly versatile genera *Parageobacillus* and *Saccharococcus*. Given the genomic versatility and extensive core genome the *Parageobacillus* and *Saccharococcus* genome dataset was evaluated for proteins of potential biotechnological value.

### Mining the *Parageobacillus* and *Saccharococcus* pan-genome for biotechnology

#### *Parageobacillus* as a source of novel antimicrobials

The emergence and rapid spread of antibiotic resistance among clinically relevant pathogens has driven the continued search for novel natural products to combat these pathogen [12]. To this extent, the geobacilli have been receiving increasing attention, with several studies identifying bacteriocins and bacteriocin-like inhibitory substances effective against a range of different pathogenic microorganisms [13–15]. antiSMASH [16] predicted on average 5.3 and 6.4 secondary metabolite biosynthetic loci in members of the genera *Parageobacillus* and *Saccharococcus*, respectively. Included among these are loci for the synthesis of metallophores (three types), betalactones (three types), betalactones (two types), a ladderane, a spore-killing factor and eight distinct bacteriocin biosynthetic loci. The latter loci were further confirmed and characterised using the BAGEL 4 [17] and RiPPMiner-Genome [18] servers.

**Fig. 4:**
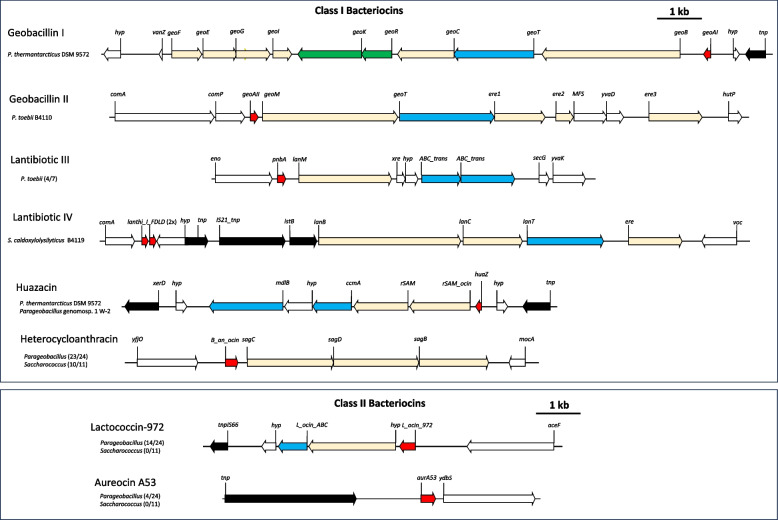
*Parageobacillus* and *Saccharococcus* antimicrobial biosynthetic loci. Schematic diagrams of the bacteriocin I and II loci present on the genomes of select *Parageobacillus* and *Saccharococcus* taxa. Genes coding for the active bacteriocin peptide are coloured in red, while those coding for accessory biosynthetic proteins, regulation and transport are shown in light yellow, green and blue, respectively. Genes coding for proteins with a potential role in self-immunity are indicated by orange arrows. A 1 kb scale bar is shown

A collection of six Class I and two Class II bacteriocins are distributed across the genome dataset. The Class I bacteriocins comprise four lantibiotic loci, a linear azole-containing peptide and a thiopeptide biosynthetic locus. The best-known *Geobacillus* antimicrobials are the lantibiotics geobacillin I and II of *G. thermodenitrificans*, effective against vancomycin-resistant *Enterococcus faecium*/methicillin-resistant *Staphylococcus aureus* and *Bacillus cereus*/*B. subtilis*, respectively [13]. The geobacillin I locus comprises ten genes, including *geoAI* which codes for the bacteriocin peptide, while the geobacillin II locus comprises three genes, with *geoAII* encoding the bacteriocin peptide [13, 15]. A complete geobacillin I locus was identified in a single taxon in our dataset, namely *P. thermantarcticus* DSM 9572 (84.6% average amino acid identity across 10 proteins; 92.9% average amino acid identity (AAI) for GeoAI bacteriocin peptide to *G. thermodenitrificans* NG80-2) (Fig. 4). Of note, 19/23 of the other *Parageobacillus* strains encode orthologues of geobacilin I self-immunity (*geoEFGI*) and two-component regulatory systems (*geoKR*) [13], suggesting they have immunity to the geobacillin I lantibiotic but are unable to produce it themselves. Only a single taxon in the dataset, *P. toebii* B4110, incorporates a geobacillin II locus (Fig. 4), which was previously shown to be more restricted in distribution than geobacillin I (only in two *G. thermodenitrificans* strains). The locus encodes all three proteins produced by the *G. thermodenitrificans* NG80-2 geobacillin II locus (99.9% average AAI). Downstream of the *P. toebii* locus are three genes coding for orthologues of erythromycin-like esterases (cd14728 – ere-like), which provide resistance to macrolides [19] and may potentially serve as a self-immunity mechanism for geobacillin II.

Two further lantibiotic biosynthetic loci types, lantibiotic III and lV were predicted on the genomes of 4/7 *P. toebii* strains and *S. caldoxylosilyticus* B4119, respectively (Fig. 4). The lantibiotic III cluster was previously identified in silico as lantibiotic cluster 4/5 [15], while the predicted bacteriocin peptide is a predicted esterase/lipase (cd00312). The lantibiotic IV cluster, novel to this study, includes genes coding for a lantibiotic dehydratase (*lanB*), cyclase (*lanC*) and ABC transporter (*lanT*), showing limited homology to the subtilin biosynthetic proteins SpaBCT of *Bacillus subtilis* (P33115-6; P39774.2; 33.5% average AAI). Two predicted FDLD family class I lanthipeptides (sharing 69.3% AAI) are encoded upstream of the other biosynthetic genes (Fig. 4).

Linear azole containing peptides (LAPs) contain heterocyclic rings of thiazole and (methyl)oxazole [20]. With the exception of *P. thermantarcticus* DSM 9572 and *S. thermophilus* DSM 4749, all examined taxa incorporate a four gene LAP biosynthetic locus coding for a cyclohydratase (*sagC*), a maturase (*sagD*) and a dehydrogenase (*sagB*) as well as a 74–113 amino acid bacteriocin peptide (71.5% AAI among the compared taxa) belonging to the heterocycloanthracin/sonorensin family (TIGR03601). Heterocycloanthracin was identified in *Bacillus cereus* and *Bacillus anthracis* [20] and sonorensin from a marine *Bacillus sonorensis* isolate [21]. Sonorensin has been shown to effective against *Listeria monocytogenes* and *Staphylococcus aureus*, with anti-biofilm activity for the latter pathogen and could be used as a food biopreservative [21]. *P. thermantarcticus* DSM 9572 and *Parageobacillus* genomosp. A W-2, incorporate a locus coding for a predicted sactibiotic (Fig. 4). Sactipeptides incorporate post-translational modifications with intramolecular bridges of cysteine sulphur to α-carbon linkages [22]. The identical 49 aa peptide in the *Parageobacillus* strains share 67.4% AAI with the huazacin peptide in *Bacillus thuringiensis* serovar *huazongensis* BGSC 4BD1 (EEM79974.1), which shows activity against the food-borne pathogen *L. monocytogenes* [23].

Two distinct class II bacteriocin loci were also identified among the studied taxa. A 123 aa peptide (98.0% AAI) present in 15/24 *Parageobacillus* taxa (all *P. thermoglucosidasius* and *P. toebii* WCH70), but absent from all *Saccharococcus* strains, is predicted to belong to the lactococcin 972 family (pfam09683), produced by *Lactococcus lactis* and active against closely related organisms [24]. The second locus encodes a 48 aa peptide and is found on the plasmids of four *P. thermoglucosidasius* strains (100% AAI). It is predicted to belong to the aureocin A53 family (NF033881), which is produced by *S. aureus* and is active against *L. monocytogenes* [25].

Another potential group of antimicrobials are lactonases, which degrade or quench *N-*acyl-homoserine lactones (AHLs) that serve as chemical signalling molecules in Gram-negative pathogens and thereby inhibit AHL-regulated functions such as the production of virulence factors and biofilms [26]. One such lactonase, GcL (WP_017434252.1) was identified in *S. caldoxylosilyticus* DSM 14590^ T^ [26]. Orthologues sharing 96.4% AAI are found in all *Parageobacillus* and five *S. caldoxylosilyticus* strains. A second predicted *N-*acyl-homoserine lactonase is found in all 35 comparator taxa and these share 68.2% AAI with the quorum quenching lactonase YntP of *B. subtilis* 168 (O34760.2). The latter lactonase inhibits streptomycin production in *Streptomyces griseus* [27]. Furthermore, orthologues (78.3% AAI) of a broad-substrate *N-*acyl-homoserine lactonase from *G. kaustophilus* HTA426 (GKL – 3OJG) [28] are encoded on 11/11 *Saccharococcus* genomes, as well as those of *P. thermantarcticus* DSM 9572^ T^ and *P. toebii* DSM 14590^ T^_._ As such, given the increasing prevalence of antimicrobial resistance, thermostable *N-*acyl-homoserine lactonases produced by *Parageobacillus* and *Saccharococcus* should receive additional attention.

#### *Parageobacillus *and *Saccharococcus* as a source of bioindustrially relevant enzymes

With a projected market share of $ 16.9 billion by 2027 [29], enzymes and in particular their thermostable counterparts, form a cornerstone of a broad range of industries, including the production of food, detergents, textiles and bioenergy [7]. Using a range of in silico tools, the *Parageobacillus* and *Saccharococcus* genomes were screened for thermostable enzymes of potential biotechnological value.

#### Carbohydrate-active enzymes

Bacteria produce a range of carbohydrate-active enzymes (CAZymes) to degrade complex carbohydrate polymers into monomeric sugars, which from a biotechnological perspective can be further fermented into biofuels and a broad range of value-added chemicals [30]. A total of 2,130 CAZymes were predicted across the 35 compared genomes (average 61 CAZymes/genome) (Additional file 2: Table S2). These were predominated by glycoside hydrolases (GH: 44.6%) that hydrolyse or rearrange glycosidic bonds in carbohydrate chains, glycosyltransferases (GT: 43.0%) that form bonds in carbohydrate chains, and carbohydrate esterases (CE: 11.5%) that deacetylate ester-substituted carbohydrates [30, 31]. Biotechnological focus is on GH and CE classes, as well as less represented polysaccharide lyases (PL: only presented on 4/35 genomes) that catalyse the non-hydrolytic cleavage of glycosidic bonds in carbohydrate chains (Additional file 2: Table S2) [30, 31]. A total of 930 GHs were identified on the 35 compared genomes, with 57 of these (6%) predicted to be extracellularly secreted. Substantially greater numbers of GHs are encoded on the genomes of members of the genus *Saccharococcus* (average GHs: 34.5/genome) than those of *Parageobacillus* (average GHs: 23.8/genome). This could largely be attributed to several strains of *S. caldoxylosilyticus*, in particular KH1-5 and KH1-6 which both code for 44 GHs (Additional file 2: Table S2).

GHs are further classified into 186 GH families [31], each with their own hydrolytic mechanism and/or substrate. The *Parageobacillus* and *Saccharococcus* GHs cover 33 distinct GH families, eight of which are predicted to be secreted extracellularly. Of these families, two are uniquely represented in the genus *Parageobacillus*, while five families are restricted to *Saccharococcus* taxa. Between seven (*P. toebii* NEB718 and *S. thermophilus* DSM 4749) and twenty-six (*S. caldoxylosilyticus* DSM 12041 and KH3-5) of the 33 GH families are encoded on each individual strain genome, with only three GH families, namely GH13, GH18 and GH23, core to all 35 compared taxa (Additional file 2: Table S2). The latter two families are involved in peptidoglycan hydrolysis and play a role in spore germination [32] and cell wall remodelling and recycling [33], respectively. The GH13 α-amylase family, which degrades starch and its derivatives (e.g. amylopectin and pullulan) [34], is the most broadly represented of all GH families among the *Parageobacillus* and *Saccharococcus* taxa, with 260 members across the 35 genomes. Being the major storage carbohydrate of terrestrial plants, starch degrading enzymes are of value in the food, fermentation and pharmaceutical industries, in particular the thermostable variants as produced by *Geobacillus* and *Parageobacillus* species [5, 6].

**Fig. 5:**
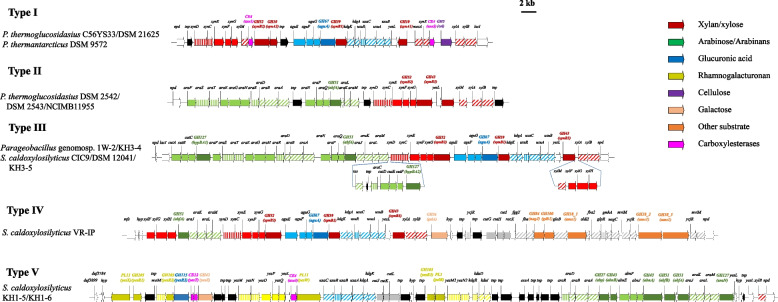
*Parageobacillus* and *Saccharococcus* Hemicellulose Utilisation (HUS) loci Schematic diagrams of the Type I-V hemicellulose utilisation loci on the genomes of select *Parageobacillus* and *Saccharococcus* taxa. Arrows corresponding to genes are coloured according to their predicted carbohydrate target. Grey arrows represent those genes coding for proteins of unrelated or unknown functions, while black arrows represent predicted transposase-associated elements. Lighter-shared arrows represent genes involved in carbohydrate transport, while cross-hatched arrows are those genes with predicted regulatory roles in carbohydrate metabolism. A 2 kb scale bar is shown

The majority of GHs encoded on the *Parageobacillus* and *Saccharococcus* genomes are involved in the degradation of lignocellulosic biomass. Lignocellulose, comprised of cellulose, hemicellulose, lignin and minor fractions of lipids, proteins, pectin and soluble sugars, forms the predominant component of plant biomass and is one of the most abundant renewable substrates on Earth [30]. In geobacilli plant biomass degradation activity can be linked to the large, highly variable Hemicellulose Utilization System (HUS) locus, which incorporates hydrolytic enzymes, sugar transport systems and carboxylesterases to completely degrade and utilise the xylose backbone, arabinose, galactose and glucuronic acid side chains and methyl or acetyl group decorations [35]. Highly variable HUS loci were found in 14/35, which could further be subdivided into five types (I-V) (Fig. 5). Type I and II are restricted to *P. thermoglucosidasius* and *P. thermantarcticus* DSM 9572 (Type I) and *P. thermoglucosidasius* only (Type II) and target xylans decorated with glucuronic acid and arabinofuranose side chains, respectively. Unique to the Type I HUS loci is a gene coding for a GH5 endoglucanase, indicating that these taxa may also target the cellulose component of biomass. Type III HUS loci were found on the genomes of the two *Parageobacillus* genomosp. A isolates and three *S. caldoxylosilyticus* strains, and are predicted to target arabinose and glucuronic acid-containing xylans. The Type IV HUS locus, unique to *S. caldoxylosilyticus* VR-IP, likely also targets this hemicellulose substrate, but further incorporates genes coding for enzymes for the hydrolysis and metabolism of galactose (GH36), mannose (GH38_1, GH38_2 and GH38_3), *N-*acetylglucosamine (GH84) and fructofuranose (GH100) [31], suggesting this strain can degrade more complex plant biomass substrates. Finally, the Type V HUS locus of *S. caldoxylosilyticus* KH1-5 and KH1-6 encodes the cellular machinery for the degradation of rhamnogalacturonan I, with pathways for the degradation of the backbone as well as arabinan and glucuronic acid side chains. This pectic polymer forms a major part of the primary cell wall and middle lamella of most higher plants [36].

The propensity of *Parageobacillus* and *Saccharococcus* taxa to degrade distinct and variously decorated plant biomass constituents offers excellent opportunities for biocomposting of plant biomass, potentially as mixed cultures [37, 38], or the production of value-added products such as oligosaccharides that could be used as prebiotics or food additives [39]. One component of plant biomass that affects the efficacy of enzymatic degradation is lignin. A lignin degrading laccase has been identified in *Geobacillus* sp. WSUCF1 (WP_011230630.1) [40]. Orthologues of this laccase are encoded on the genomes all 35 studied taxa (61.4% AAI), suggesting that they further incorporate the machinery to degrade this plant biomass constituent.

#### Lipases, carboxylesterases and proteases

Thermostable lipases and carboxylesterases are of growing interest in the food, pharmaceutical and fine-chemical industries, where their products of hydrolysis can be used for the synthesis of various chemicals [2, 6]. Where lipases degrade water-insoluble long chain triglycerides, carboxylesterases hydrolyse ester bonds in shorter chain acyl derivatives [6]. Comparison of the *Parageobacillus* and *Saccharococcus* proteomes against the Lipase Engineering Database (LED) [41] identified orthologues for twenty-four distinct homologous family groups (Additional file 2: Table S2). Of these, thirteen constituted alpha/beta hydrolases (abhydrolases – cl 21,494) for which no clear substrate/activity could be identified, while five distinct acetyl esterases are predicted to contribute to the removal of acetyl groups from lignocellulosic components (xylan and rhamnogalacturonan). Three distinct carboxylesterases are encoded on the genomes. *p*-Nitrobenzyl esterases need to be removed from oral beta-lactam antibiotics for their final synthesis, and the *p-*nitrobenzyl esterase (PbnA) of *B. subtilis* is effective in this activity [42]. Orthologues of this enzyme (P37967.2; 44.2% AAI) are present in 11/11 *Saccharococcus* strains and *P. thermantarcticus* DSM 9572. Orthologues of two characterised carboxylesterases from *Geobacillus stearothermophilus* (Est30; Pdb = 1TQH; 90% AAI) and *G. thermodenitrificans* CMB-A2 (EstGtA2; AEN92268.1; 72% AAI) are present in all 35 analysed taxa. Both of these thermostable enzymes show activity against *p*-nitrophenyl esters of different chain length [43, 44]. All 35 *Parageobacillus* and *Saccharococcus* taxa also encode orthologues of a lysophospholipase (YpA; COG 2267) as well as two distinct copies of GDSL-like lipases (pfam 13,472). However, the target triglycerides would need to be determined.

Microbial proteases and peptidases, in particular their thermostable counterparts, have a broad range of applications including the treatment of leather, as additives in detergents and in the food industry [2, 6]. Comparison of the proteome datasets against the MEROPS database [45] identified 4,765 distinct protein orthologues encoded on the 35 genomes. On average, slightly more (138) are encoded on the *Saccharococcus* genomes than on the *Parageobacillus* genomes (135), while 130 are encoded on the genome of *G. thermodenitrificans* DSM 465^ T^. The highest number of proteases/peptidases are encoded on the genome of *S. caldoxylosilyticus* B4119 (152) (Additional file 2: Table S2). The proteases/peptidases belong to 40 and 66 distinct MEROPS clans and families, respectively, with the highest numbers of families represented in *Saccharococcus* genomosp. A NUB3621 (60). The proteases/peptidases can be subdivided into 212 orthogroups, 91 of which (43%) are core to all compared taxa, while 33 (16%) occur only in a single taxon. A total of 40 and 21 protease/peptidase orthogroups are unique to either the genus *Parageobacillus* or *Saccharococcus*, respectively. Only a small proportion (23/212) of the protease/peptidase orthogroups are secreted extracellularly, with six each of these unique to *Parageobacillus* and *Saccharococcus* taxa, respectively.

*Parageobacillus* genomosp. nov. A KH3-4 and W-2 as well as *Saccharococcus* genomosp. nov. A NUB3621 (two copies) produce a predicted neutral thermolysin protease sharing 68.2% AAI (range 51.6–82.7%) with thermolysin from *G. stearothermophilus* (P43133.1). The latter protease (NprS) is commercially used to produce precursors for the artificial sweetener aspartame [46]. Serine proteases, particularly those of the subtilisin superfamily (S8), have a broad range of applications in the food, cosmetics and detergent industries, and in the treatment of sewage [47]. A total of 171 S8 family proteases are encoded across the *Parageobacillus/Saccharococcus* genomes*,* belonging to 16 distinct orthogroups (12/16 extracellularly secreted). Orthogroups of five and one subtilisin protease are unique to single strains of *Parageobacillus* and *Saccharococcus*, respectively, while a further three orthogroups are represented in *Parageobacillus* species only. While the S8 proteases in these taxa share between 27.1 and 43.6% AAI with subtilisin J of *G. stearothermophilus* NCIMB 10278 (P29142.1; 27), the S8 protease orthogroups in this study share < 50% AAI among them, indicating a broad underexplored set of proteases of potential biotechnological value among the genera *Parageobacillus* and *Saccharococcus*.

#### Enzymes for the molecular laboratory

Thermostable DNA-active enzymes encompass an expanding toolkit for numerous conventional molecular biotechnology applications, including PCR, genetic engineering, DNA sequencing, diagnostics and synthetic biology [5]. Several thermostable DNA polymerases have been derived and commercially developed from *Geobacillus* spp., most notably the Bst DNA polymerase, a family A DNA polymerase I with 5'-3' exonuclease activity isolated from *G. stearothermophillus* GIM1.543 [48]. All strains analysed possessed DNA polymerases of the families A (DNA PolI), C (DNA PolIII—α, τγ, δ, δ′ and β subunits), Y (DNA PolIV) and X (DNA PolX), represented by one orthogroup each (Additional file 2: Table S3). In addition, a putative DNA polymerase family B (PolB) orthologue, is encoded on the genomes of *P. thermantarcticus* DSM 9572^ T^ and *P. thermoglucosidasius* DSM 21625. In addition to the DinB DNA polymerase IV orthologues (74.2% AAI; range 52.0–100%) encoded by all strains analysed, two *P. toebii* and eight *S. caldoxylosilyticus* strains encode putative UmuC DNA polymerase family Y (DNA PolV) orthologues (81.8% AAI) involved in UV-dependent and chemically-induced mutagenesis [49]. These polymerases may have application in inducing random mutagenesis for the purpose of directed evolution [50].

Thermostable restriction enzymes and their associated modification (RM) systems are used in various generic engineering strategies, sequencing and diagnostics [51]. Comparison to the REBASE database [52] identified 61 orthogroups incorporating restriction-modification (RM) components (Additional file 2: Table S4). These included twenty-seven Type I, eighteen Type II, nine Type III and six Type IV putative RM components. Most (59/61) of the identified RM components are encoded on the genomes of *Parageobacillus* spp., 43 of which are unique to the genus. Of these, twelve and twenty-three are specific to *P. thermoglucosidasius* and *P. toebii*, respectively. *Saccharococcus* genomes only encode 19/61 of the RM components, three of which are unique to *S. caldoxylosilyticus* strains. On average, ~ 7 and 3 RM components are encoded on the genomes of *Parageobacillus* and *Saccharococcus*, respectively, suggesting they, and in particular the former genus, represent a rich source for novel thermostable RM enzymes.

In addition to the native role CRISPR-Cas systems play in preventing foreign plasmid and nucleic acid transfer in prokaryotes [53], modified CRISPR-Cas systems have also been employed in various biotechnological and biomedical applications through targeted genome editing and gene regulation [54]. Recently, several *Geobacillus* Cas proteins have also received attention due to their thermostability and greater specificities when compared to the more frequently utilised mesophilic Cas9 systems [55]. Using CRISPRCasFinder [56], 34 distinct orthogroups were identified as Cas proteins of type I and type III CRISPR-Cas systems (Additional file 2: Table S5). Substantially more Cas proteins were identified in *P. thermoglucosidasius* (average Cas proteins: 16.96/genome) compared to *Saccharococcus* spp. (average Cas proteins: 6.1/ genome).

### Whole-cell biotechnological applications for *Parageobacillus* and *Saccharococcus*

#### Applications of *Parageobacillus* and *Saccharococcus* in bioremediation

**Fig. 6:**
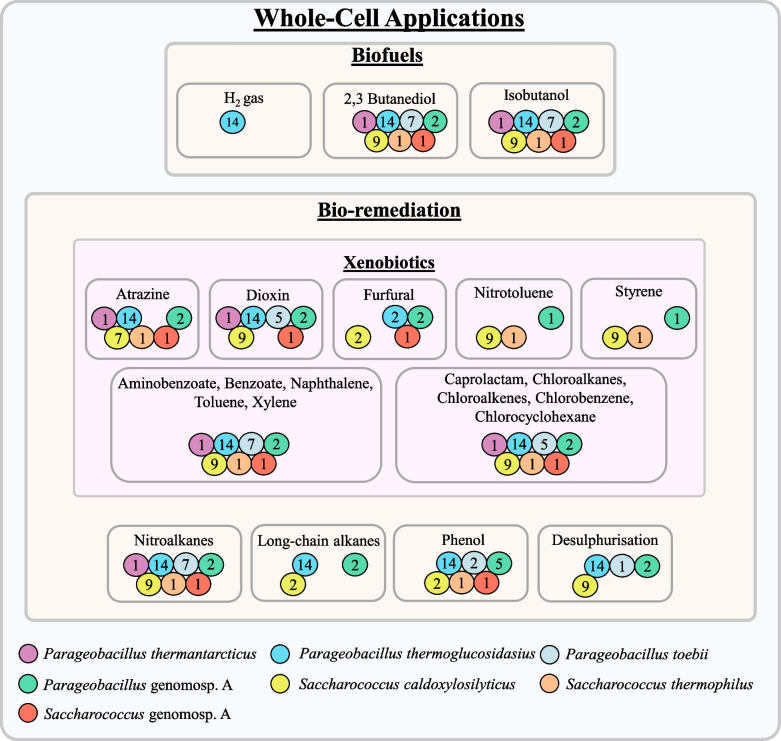
Schematic diagram showing potential whole-cell applications of the genera *Parageobacillus* and *Saccharococcus*. Coloured balls represent species containing at least one gene orthologue or pathway component for each respective system

Aside from the biotechnological potential of their enzymes, there has also been extensive interest in whole cell biocatalysis with thermophilic Geobacilli (Fig. 6) [2]. Numerous *Geobacillus* (and *Parageobacillus*) strains have been investigated for their applicability towards various bioremediation applications, including degradation of xenobiotics, phenols and in particular long chain- and aromatic-hydrocarbons and petroleum hydrocarbons [6].

Analysis of the comparator protein dataset identified 84 distinct orthogroups associated with degradation of various xenobiotic compounds (Additional file 2: Table S6). The genomes of *P*. *thermoglucosidasius* taxa typically encode substantially more orthologues (61.4/genome) than either *Saccharococcus* spp. (43.6/genome) or *P. toebii* (28.9/genome; Fig. 6). The highest number of proteins involved in xenobiotic degradation occur in *P. thermoglucosidasius* 23.6 (69).

Phenol *meta*-cleavage pathway degradation loci (a twelve gene chromosomal and ten gene plasmid locus) have previously been identified in the genus *Parageobacillus* [57]. The full chromosomal phenol degradation operon is conserved among all *P. thermoglucosidasius* strains, 2/7 *P. toebii*, both *Parageobacillus* genomosp. A strains and 5/8 *S. caldoxylosilyticus* strains. (Additional file 2: Table S6). Furthermore, 8/14 *P. thermoglucosidasius* strains carry the complete plasmid-bound locus.

Crude and refined petroleum fractions may contain or release (upon combustion) high levels of organosulphur compounds, which are resistant to degradation and hazardous to the environment [58]. Consequently, biological means of reducing levels of organosulfur compounds either preventatively in processed petroleum products, or in the remediation of polluted systems, is desirable. Various thermophilic taxa have been observed capable of catabolising sulphur-rich petroleum compounds, including members of the genus *Parageobacillus* [58]. Thirty distinct orthogroups were associated with sulphur metabolism. Three desulphurization-associated gene clusters (1, 2 and 3; Additional file 2: Table S6), incorporating distinct monooxygenases, have previously been described in *Parageobacillus thermoglucosidasius* [58]. The genomes of all *P. thermoglucosidasius* and two *S. caldoxylosilyticus* strains incorporate all three complete desulphurisation clusters, while those of 7/9 *S. caldoxylosilyticus* strains and the two *Parageobacillus* genomosp. A isolates incorporate complete desulphurisation clusters 2 and 3 (Fig. 6). *S. caldoxylosilyticus* VR-IP and *P. toebii* WCH70 harboured only a complete desulphurisation cluster 3, while none were observed in the other *P. toebii* strains (Fig. 6).

Long-chain alkanes form a major component of crude oils. Several studies have identified the presence and activity of genes associated with variable length long-chain alkane catabolism in *Geobacillus* and *Parageobacillus* taxa [59]. Orthologues of LadAα (ART30136: 66.62–67.69% AAI range) and LadAβ (ART30139: 70.85–71.86% AAI range) and LadB (ART30142: 61.81–66.54% AAI range) that contribute to C_10_-C_30_ *n*-alkane utilisation in *P. toebii* B1024 [59] are encoded on the genomes of all *P. thermoglucosidasius* strains analysed, *Saccharococcus* genomosp. A KH3-5, *S. caldoxylosilyticus* ER4B. Both LadA orthologues, but no LadB orthologues are present in *Parageobacillus* genomosp. A KH3-4 and W-2 (Fig. 6; Additional file 2: Table S6). In accompaniment, at least one putative aldehyde dehydrogenase (ABO68462; 78.89–94.52% AAI range) and three alcohol dehydrogenase orthologues (ABO66657, ABO67118 and ABO68223; 76.77–79.42%, 79.76–86.10% and 86.7–91.3% AAI ranges, respectively), assumed to participate in LadA-initiated metabolism of long-chain alkanes in *G. thermodenitrificans* NG80-2 [59], were detected across all strains except *S. thermophilus* DSM 4749, which did not encode orthologues of ABO68462 or ABO67118.

Nitroalkanes are another group of highly recalcitrant compounds, utilized as fuels, solvents, herbicides and pesticides which are also toxic and carcinogenic [60]. Recently, three nitroalkane oxidizing enzymes (WP_064553126, WP_064551563 WP_064551165) were shown to variably degrade nitropropane and nitroethane in *Parageobacillus* genomosp. A W-2 [60]. Orthologues of each enzyme (Gt2929, Gt1378 and Gt1208; 55.20–100%, 76.96–100% and 88.73–100% AAI ranges, respectively) are encoded on the genomes of 34, 33 and all 35 of the analysed strains, respectively (Fig. 6; Additional file 2: Table S6).

#### *Parageobacillus* as a producer of green energy

In part due to its capacity for biomass degradation, as well as its fermentation pathways, *P. thermoglucosidasius* has received extensive interest for the production of biofuels (Fig. 6). In particular, ethanol production has been widely researched, but as a mixed acid fermenter with limited ethanol tolerance, metabolic engineering of this species is required [61].

Another *P. thermoglucosidasius* fermentation product of biotechnological interest is isobutanol, which can serve as biofuel, fuel additive or as a primer for the production of chemicals [6, 62]. The final step in isobutanol formation from isobutyraldehyde involves an isobutayraldehyde dehydrogenase, with two putative enzymes (AdhA and Geoth_3823) identified in *P. thermoglucosidasius* C56YS93 [62]. Orthologues of both enzymes, sharing 95.7% and 97.3%, are encoded on the genomes of all 35 and 33/35 comparator strains, respectively (Fig. 6), suggesting members of both genera could serve as targets for metabolic engineering for isobutanol production. *P. thermoglucosidasius* also produces 2,3-butanediol (2,3-BDO), which can be used as liquid fuel, fuel additive or chemically modified to produce high octane isomers for use in aviation fuels [6, 63]. Orthologues of one key enzyme involved in 2,3-BDO synthesis identified in *P. thermoglucosidasius* NCIMB 11955 [63], namely acetolactate synthase (ALS), were observed in all compared taxa (93.1% AAI; Fig. 6). The final enzyme in 2,3-BDO synthesis, butanediol dehydrogenase (BDH), was restricted to the genus *Parageobacillus* (97.9%), with a single copy encoded on the genome of 23/24 taxa, with the exception of *P. toebii* WCH70, where two copies exist (96.6% amino acid identity between copies).

Recent interest has focused on the production of hydrogen gas, an environmentally friendly and sustainable alternative energy carrier, from carbon monoxide-containing waste gases by *P. thermoglucosidasius* [64]. This biological water–gas shift reaction (WGS) involves an enzyme complex comprising a carbon-monoxide dehydrogenase (CODH) and hydrogen-evolving hydrogenase [64]. Previous analyses showed the CODH-hydrogenase locus to be restricted to *P. thermoglucosidasius*. Analysis of our annotated dataset showed homologous loci in all fourteen *P. thermoglucosidasius* taxa, with the CODH proteins CooCSF and hydrogenase proteins PhcABCDEFGHIJKL sharing 99.5% and 99.1% AAI, respectively among these taxa, while no orthologues were found in any other *Parageobacillus* or *Saccharococcus* taxa (Fig. 6). However, a recent study identified the CODH-hydrogenase locus on the genome of *Parageobacillus* sp. G301 [65]. ANI and dDDH values (97.24% and 76.3%, respectively with *P. toebii* DSM 14590^ T^) indicate that this strain belongs to the species *P. toebii*, and the CODH and hydrogenase proteins share an average AAI of 91.5% and 91.2% with those of the fourteen *P. thermoglucosidasius* taxa. As such, broader evaluation of the genera *Parageobacillus* and *Saccharococcus* for hydrogen-evolving systems of potential biotechnological value is warranted.

## Conclusions

Phylogenomic analysis delineates *Parageobacillus* and *Saccharococcus* as two distinct genera, both of which present open pan-genomes. *P. toebii* in particular presents the greatest potential for novel gene accrual within *Parageobacillus*. Plasmids, bacteriophages and transposable elements are key drivers of genomic and functional, diversification of these genera. Both *Parageobacillus* and *Saccharococcus* harbour a wealth of biotechnological potential including potential novel antimicrobials and a range of thermostable enzymes. Functional and in vivo analyses of the novel antimicrobial peptides should serve to validate the potential of the studied taxa to contribute towards combatting antibiotic-resistant target bacteria. Similarly, the broad range of carbohydrate-, protein- and lipid-active enzymes, identified here and in previous studies, should be evaluated to expand the current repertoire of thermostable enzymes for a wide array of biotechnological applications. Our analyses have also further highlighted the potential for members of both *Parageobacillus* and *Saccharococcus* in a broad spectrum of whole-cell applications, including bioremediation of various xenobiotic compounds and environmental pollutants, the degradation of lignocellulosic biomass to generate various value-added products, as well as the use of these taxa to contribute towards the green energy market. Given the extensive genomic variability and the potential biotechnological pathways and enzyme complement, additional discovery and characterization, both genomic and functional, of novel *Parageobacillus* and *Saccharococcus* isolates will continue to expand the biotechnological toolkit of these intriguing genera.

## Methodology

### Genome assembly and annotation

The publicly available genome sequences of thirty-four *Parageobacillus* taxa*, Saccharococcus thermophilus* DSM 4749^ T^ and *G. thermodenitrificans* DSM 465^ T^ (used for comparative and outgroup purposes) were obtained from the NCBI genome assembly database [66]. Average Nucleotide Identity (ANI) values of all draft genomes were calculated with the OAT tool v. 0.9.1 [67]. The genome assemblies were subsequently improved using the MeDuSa genome scaffolder v. 1.6 [68], where the genome of the taxon sharing the highest ANI value (complete genome) was used as reference genome. All genomes were structurally annotated using Prodigal v.2.6.3 [69] and the proteome datasets were functionally annotated (and assigned to COG categories) using eggnog-mapper v. 2.1.12 [70] against the eggNOG v. 5.0 database [71]. The subcellular localisations of all proteins encoded on each genome were determined using PSORTb v. 3.0.3 [72]. Plasmids and transposable elements were identified on the basis of the eggNOG annotations, while phage elements were identified using the PHASTER server [73].

Biotechnologically relevant enzymes were identified and characterised using several pipelines. Secondary metabolite biosynthetic loci were identified using antiSMASH v. 7.0.1 [16] and further confirmed and characterised using the BAGEL 4 [17] and RiPPMiner-Genome [18] servers. CAZYmes were predicted from the protein datasets for each genome using the HMMer, Hotpep and DIAMOND tools of DbCAN3 [74] against the CAZYme database [31], where only those predictions made by ≥ 2 tools were considered as positive hits. Proteases/peptidases and lipases were identified and characterised by aligning the proteome datasets for each compared *Parageobacillus* and *Saccharococcus* strain against the MEROPS v. 11.0 database [45] and the Lipase Engineering Database (LED) v. 4.1.0 [41], respectively, using DIAMOND v. 2.1.8 [75]. CRISPR-Cas associated proteins were predicted through the CRISPRCasFinder tool v. 1.1.2—I2BC [56]. Other proteins of potential biotechnological relevance were identified by localized Blast analyses and alignment using Bioedit v. 7.7.1 [76]. Restriction-modification systems were tentatively identified on the basis of the eggnog-mapper annotations and confirmed through Blastp analysis against the REBASE database [52].

### Phylogenomic analyses

The proteome datasets for each comparator strain (and *G. thermodenitrificans* DSM 465^ T^ as outgroup) were compared and clustered into their orthologous groups using Orthofinder v. 2.5.5 [77]. Single copy orthologous (SCO) proteins conserved among all taxa (1,784 SCOs) were individually aligned using the M-Coffee implementation of T-Coffee v. 13.46.0.919e8c6b [78], concatenated and poorly aligned blocks were removed using GBlocks v. 0.91b [79]. The trimmed concatenated alignment was used to construct a maximum likelihood (ML) phylogeny using IQ-Tree v. 2.2.0 [80], with the optimal evolutionary model predicted using ModelFinder [81]. Branch support was provided using ultrafast bootstrap (UFBoot2) analysis (n = 1,000 replicates) [82]. Support for the core genome phylogeny and species delineation was provided by calculating the Average Nucleotide Identity (ANI) values with the OAT tool v. 0.9.1 [67] and digital DNA-DNA hybridization values (dDDH) were determined using the Genome-to-Genome Distance Calculator (GGDC 3.0) [83], where taxa sharing OrthoANI values > 96% and dDDH values > 70% were considered to belong to the same species [8, 67, 83].

### Pan-genome analyses

The Orthofinder output was used to identify the core (conserved among all taxa), accessory (shared by several but not all compared strains) and unique (to a single taxon) proteome fractions of the compared *Parageobacillus* and *Saccharococcus* taxa. The presence (1) or absence (0) of each orthogroup was scored and the pan-genome of different datasets (*Parageobacillus/Saccharococcus; P. thermoglucosidasius/P. toebii/S. caldoxylosilyticus*) were used to determine the pan-genome using the bacterial pan-genome analysis (BPGA) pipeline [84] and extrapolated (to 100 genomes/per set of taxa) using PanGP [85]. The functions of the core, accessory and unique pan-genome fractions were determined by comparison of the pan-genome element-specific proteome datasets against the eggNOG v. 5.0 database [71] using eggnog-mapper v. 2.1.12 [70].

### Supplementary Information

Supplementary Material 1.

Supplementary Material 2.

## Data Availability

The genome datasets analysed in this study are available at the NCBI genome assembly database (https://www.ncbi.nlm.nih.gov/datasets/). All data generated during this study is included in the article and its additional files.

## References

[1] Zeigler DR. The *Geobacillus* paradox: why is a thermophilic bacterial genus so prevalent on a mesophilic planet? Microbiology. 2014;160(1):1–11. 24085838 10.1099/mic.0.071696-0

[2] Hussein AH, Lisowska BK, Leak DJ. The genus *Geobacillus* and their biotechnological potential. Adv Appl Microbiol. 2015;92:1–48. 26003932 10.1016/bs.aambs.2015.03.001

[3] Ash C, Farrow JA, Wallbanks S, Collins MD. Phylogenetic heterogeneity of the genus *Bacillus* revealed by comparative analysis of small-subunit-ribosomal RNA sequences. Lett Appl Microbiol. 1991;13(4):202–6. 10.1111/j.1472-765X.1991.tb00608.x

[4] Parte AC, Carbasse JS, Meier-Kolthoff JP, Reimer LC, Göker M. List of Prokaryotic Names with Standing in Nomenclature (LPSN) moves to the DSMZ. Int J Syst Evol Microbiol. 2020;70(11):5607–12. 32701423 10.1099/ijsem.0.004332 PMC7723251

[5] Najar IN, Thakur N. A systematic review of the genera *Geobacillus* and *Parageobacillus*: their evolution, current taxonomic status and major applications. Microbiology. 2020;166(9):800–16. 32744496 10.1099/mic.0.000945

[6] Novik G, Savich V, Meerovskaya O. *Geobacillus* bacteria: potential commercial applications in industry, bioremediation and bioenergy production. In: Mishra M, editor. Growing and handling of bacterial cultures. London: IntechOpen; 2018. p. 1–36.

[7] Kumar S, Dangi AK, Shukla P, Baishya D, Khare SK. Thermozymes: adaptive strategies and tools for their biotechnological applications. Bioresour Technol. 2019;278:372–82. 30709766 10.1016/j.biortech.2019.01.088

[8] Aliyu H, Lebre P, Blom J, Cowan D, De Maayer P. Phylogenomic re-assessment of the thermophilic genus *Geobacillus*. Syst Appl Microbiol. 2016;39(8):527–33. 27726901 10.1016/j.syapm.2016.09.004

[9] Nystrand R. *Saccharococcus thermophilus* gen. nov., sp. nov. isolated from beet sugar extraction. Syst Appl Microbiol. 1984;5:204–19. 10.1016/S0723-2020(84)80021-1

[10] Ahmad S, Scopes RK, Rees GN, Patel BK. *Saccharococcus caldoxylosilyticus* sp. nov., an obligately thermophilic, xylose-utilizing, endospore-forming bacterium. Int J Syst Evol Microbiol. 2000;50:517–23. 10758855 10.1099/00207713-50-2-517

[11] De Maayer P, Chan WY, Rubagotti E, Venter SN, Toth IK, Birch PRJ, et al. Analysis of the *Pantoea ananatis* pan-genome reveals factors underlying its ability to colonize and interact with plant, insect and vertebrate hosts. BMC Genomics. 2014;15:1–14. 24884520 10.1186/1471-2164-15-404 PMC4070556

[12] Rossiter SE, Fletcher MH, Wuest WM. Natural products as platforms to overcome antibiotic resistance. Chem Rev. 2017;117(19):12415–74. 28953368 10.1021/acs.chemrev.7b00283 PMC5869711

[13] Garg N, Tang W, Goto Y, Nair SK, van der Donk WA. Lantibiotics from *Geobacillus thermodenitrificans*. Proc Natl Acad Sci. 2012;109(14):5241–6. 22431611 10.1073/pnas.1116815109 PMC3325677

[14] Zebrowska J, Witkowska M, Struck A, Laszuk PE, Raczuk E, Ponikowska M, et al. Antimicrobial potential of the genera *Geobacillus* and *Parageobacillus*, as well as endolysins biosynthesized by their bacteriophages. Antibiotics. 2022;11(12):242. 35203843 10.3390/antibiotics11020242 PMC8868475

[15] Egan K, Field D, Ross RP, Cotter PD, Hill C. *In silico* prediction and exploration of potential bacteriocin gene clusters within the bacterial genus *Geobacillus*. Front Microbiol. 2018;9:2116. 30298056 10.3389/fmicb.2018.02116 PMC6160750

[16] Blin K, Shaw S, Augustijn HE, Reitz ZL, Biermann F, Alanjary M, et al. antiSMASH 7.0: new and improved predictions for detection, regulation, chemical structures and visualisation. Nucleic Acids Res. 2023;51(W1):W46-50. 37140036 10.1093/nar/gkad344 PMC10320115

[17] van Heel AJ, de Jong A, Song C, Viel JH, Kok J, Kuipers OP. BAGEL4: a user-friendly web server to thoroughly mine RiPPs and bacteriocins. Nucleic Acids Res. 2018;46(W1):W278–81. 29788290 10.1093/nar/gky383 PMC6030817

[18] Agrawal P, Amir S, Barua D, Mohanty D. RiPPMiner-Genome: a web resource for automated prediction of crosslinked chemical structures of RiPPs by genome mining. J Mol Biol. 2021;433(11):166887. 33972022 10.1016/j.jmb.2021.166887

[19] Zieliński M, Park J, Sleno B, Berghuis AM. Structural and functional insights into esterase-mediated macrolide resistance. Nat Commun. 2021;12(1):1732. 33741980 10.1038/s41467-021-22016-3 PMC7979712

[20] Haft DH. A strain-variable bacteriocin in *Bacillus anthracis* and *Bacillus cereus* with repeated Cys-Xaa-Xaa motifs. Biol Direct. 2009;4:15. 19383135 10.1186/1745-6150-4-15 PMC2674419

[21] Chopra L, Singh G, Kumar Jena K, Sahoo DK. Sonorensin: a new bacteriocin with potential of an anti-biofilm agent and a food biopreservative. Sci Rep. 2015;5(1):13412. 26292786 10.1038/srep13412 PMC4544038

[22] Mathur HC, Rea MD, Cotter P, Hill C, Paul Ross R. The sactibiotic subclass of bacteriocins: an update. Curr Protein Pept Sci. 2015;16(6):549–58. 26031307 10.2174/1389203716666150515124831

[23] Hudson GA, Burkhart BJ, Di Caprio AJ, Schwalen CJ, Kille B, Pogorelov TV, et al. Bioinformatic mapping of radical S-adenosylmethionine-dependent ribosomally synthesized and post-translationally modified peptides identifies new Cα, Cβ, and Cγ-linked thioether-containing peptides. J Am Chem Soc. 2019;141(20):8228–38. 31059252 10.1021/jacs.9b01519 PMC6622460

[24] Martínez B, Fernández M, Suárez JE, Rodríguez A. Synthesis of lactococcin 972, a bacteriocin produced by *Lactococcus lactis* IPLA 972, depends on the expression of a plasmid-encoded bicistronic operon. Microbiology. 1999;145(11):3155–61. 10589723 10.1099/00221287-145-11-3155

[25] Netz DJA, Pohl R, Beck-Sickinger AG, Selmer T, Pierik AJ, Bastos M do C de F, et al. Biochemical characterisation and genetic analysis of Aureocin A53, a new, atypical bacteriocin from *Staphylococcus aureus*. J Mol Biol. 2002;319:745–56. 12054867 10.1016/S0022-2836(02)00368-6

[26] Bergonzi C, Schwab M, Elias M. The quorum-quenching lactonase from *Geobacillus caldoxylosilyticus*: purification, characterization, crystallization and crystallographic analysis. Acta Crystallogr Sect F Struct Biol Commun. 2016;72(9):681–6. 27599858 10.1107/S2053230X16011821 PMC5012207

[27] Schneider J, Yepes A, Garcia-Betancur JC, Westedt I, Mielich B, López D. Streptomycin-induced expression in *Bacillus subtilis* of YtnP, a lactonase-homologous protein that inhibits development and streptomycin production in *Streptomyces griseus*. Appl Environ Microbiol. 2012;78(2):599–603. 22101040 10.1128/AEM.06992-11 PMC3255736

[28] Chow JY, Xue B, Lee KH, Tung A, Wu L, Robinson RC, et al. Directed evolution of a thermostable quorum-quenching lactonase from the amidohydrolase superfamily. J Biol Chem. 2010;285(52):40911–20. 20980257 10.1074/jbc.M110.177139 PMC3003391

[29] Enzymes market by product type (Industrial enzymes and specialty enzymes), Source (Microorganism, plant, and animal), Type, Industrial enzyme application, Specialty enzymes application and Region - Global forecast to 2027. https://www.marketsandmarkets.com/Market-Reports/enzyme-market-46202020. Accessed 15 Feb 2020.

[30] Chettri D, Verma AK, Verma AK. Innovations in CAZyme gene diversity and its modification for biorefinery applications. Biotechnol Rep. 2020;28:e00525. 10.1016/j.btre.2020.e00525 PMC7490808 32963975

[31] Drula E, Garron ML, Dogan S, Lombard V, Henrissat B, Terrapon N. The carbohydrate-active enzyme database: functions and literature. Nucleic Acids Res. 2022;50(D1):D571–7. 34850161 10.1093/nar/gkab1045 PMC8728194

[32] Kodama T, Takamatsu H, Asai K, Kobayashi K, Ogasawara N, Watabe K. The *Bacillus subtilis yaaH* gene is transcribed by SigE RNA polymerase during sporulation, and its product is involved in germination of spores. J Bacteriol. 1999;181(15):4584–91. 10419957 10.1128/JB.181.15.4584-4591.1999 PMC103590

[33] Byun B, Mahasenan KV, Dik DA, Marous DR, Speri E, Kumarasiri M, et al. Mechanism of the *Escherichia coli* MltE lytic transglycosylase, the cell-wall-penetrating enzyme for Type VI secretion system assembly. Sci Rep. 2018;8(1):4110. 29515200 10.1038/s41598-018-22527-y PMC5841429

[34] Stam MR, Danchin EGJ, Rancurel C, Coutinho PM, Henrissat B. Dividing the large glycoside hydrolase family 13 into subfamilies: towards improved functional annotations of α-amylase-related proteins. Protein Eng Des Sel. 2006;19(12):555–62. 17085431 10.1093/protein/gzl044

[35] De Maayer P, Brumm PJ, Mead DA, Cowan DA. Comparative analysis of the *Geobacillus* hemicellulose utilization locus reveals a highly variable target for improved hemicellulolysis. BMC Genomics. 2014;15(1):1–7. 25273399 10.1186/1471-2164-15-836 PMC4194401

[36] Kaczmarska A, Pieczywek PM, Cybulska J, Zdunek A. Structure and functionality of Rhamnogalacturonan I in the cell wall and in solution: a review. Carbohydr Polym. 2022;278: 118909. 34973730 10.1016/j.carbpol.2021.118909

[37] Wang M, Zhu H, Kong Z, Li T, Ma L, Liu D, et al. Pan-genome analyses of *Geobacillus* spp. reveal genetic characteristics and composting potential. Int J Mol Sci. 2020;21(9):3393. 32403359 10.3390/ijms21093393 PMC7246994

[38] Sarkar S, Banerjee R, Chanda S, Das P, Ganguly S, Pal S. Effectiveness of inoculation with isolated *Geobacillus* strains in the thermophilic stage of vegetable waste composting. Bioresour Technol. 2010;101(8):2892–5. 20036532 10.1016/j.biortech.2009.11.095

[39] Placier G, Watzlawick H, Rabiller C, Mattes R. Evolved β-galactosidases from *Geobacillus stearothermophilus* with improved transgalactosylation yield for galacto-oligosaccharide production. Appl Environ Microbiol. 2009;75(19):6312–21. 19666723 10.1128/AEM.00714-09 PMC2753058

[40] Rai R, Bibra M, Chadha BS, Sani RK. Enhanced hydrolysis of lignocellulosic biomass with doping of a highly thermostable recombinant laccase. Int J Biol Macromol. 2019;137:232–7. 31260768 10.1016/j.ijbiomac.2019.06.221

[41] Fischer M, Pleiss J. The Lipase Engineering Database: a navigation and analysis tool for protein families. Nucleic Acids Res. 2003;31(1):319–21. 12520012 10.1093/nar/gkg015 PMC165462

[42] Zock J, Cantwell C, Swartling J, Hodges R, Pohl T, Sutton K, et al. The *Bacillus subtilis pnbA* gene encoding *p-*nitrobenzyl esterase: cloning, sequence and high-level expression in *Escherichia coli*. Gene. 1994;151(1):37–43. 7828905 10.1016/0378-1119(94)90630-0

[43] Montoro-García S, Martínez-Martínez I, Navarro-Fernández J, Takami H, García-Carmona F, Sánchez-Ferrer Á. Characterization of a novel thermostable carboxylesterase from *Geobacillus kaustophilus* HTA426 shows the existence of a new carboxylesterase family. J Bacteriol. 2009;191(9):3076–85. 19304850 10.1128/JB.01060-08 PMC2681792

[44] Charbonneau DM, Meddeb-Mouelhi F, Beauregard M. A novel thermostable carboxylesterase from *Geobacillus thermodenitrificans*: evidence for a new carboxylesterase family. J Biochem. 2010;148(3):299–308. 20587647 10.1093/jb/mvq064

[45] Rawlings ND, Barrett AJ, Thomas PD, Huang X, Bateman A, Finn RD. The MEROPS database of proteolytic enzymes, their substrates and inhibitors in 2017 and a comparison with peptidases in the PANTHER database. Nucleic Acids Res. 2018;46(D1):D624–32. 29145643 10.1093/nar/gkx1134 PMC5753285

[46] Ke Q, Chen A, Minoda M, Yoshida H. Safety evaluation of a thermolysin enzyme produced from *Geobacillus stearothermophilus*. Food Chem Toxicol. 2013;59:541–8. 23831195 10.1016/j.fct.2013.06.046

[47] Falkenberg F, Voß L, Bott M, Bongaerts J, Siegert P. New robust subtilisins from halotolerant and halophilic *Bacillaceae*. Appl Microbiol Biotechnol. 2023;107(12):3939–54. 37160606 10.1007/s00253-023-12553-w PMC10238314

[48] Oscorbin I, Filipenko M. Bst polymerase — a humble relative of Taq polymerase. Comput Struct Biotechnol J. 2023;21:4519–35. 37767105 10.1016/j.csbj.2023.09.008 PMC10520511

[49] Timinskas K, Venclovas Č. New insights into the structures and interactions of bacterial Y-family DNA polymerases. Nucleic Acids Res. 2019;47(9):4383–405. 10.1093/nar/gkz198 PMC6511836 30916324

[50] Labrou NE. Random mutagenesis methods for in vitro directed enzyme evolution. Curr Protein Pept Sci. 2010;11(1):91–100. 20201809 10.2174/138920310790274617

[51] Sharma P, Kumar R, Capalash N. Restriction enzymes from thermophiles. In: Satyanarayana T, Littlechild J, Kawarabayasi Y, editors. Thermophilic microbes in environmental and industrial biotechnology: biotechnology of thermophiles. Dordrecht: Springer, Netherlands; 2013. p. 611–47.

[52] Roberts RJ, Vincze T, Posfai J, Macelis D. REBASE—a database for DNA restriction and modification: enzymes, genes and genomes. Nucleic Acids Res. 2015;43(D1):D298–9. 25378308 10.1093/nar/gku1046 PMC4383893

[53] Westra ER, Staals RHJ, Gort G, Høgh S, Neumann S, de la Cruz F, et al. CRISPR-Cas systems preferentially target the leading regions of MOBF conjugative plasmids. RNA Biol. 2013;10(5):749–61. 23535265 10.4161/rna.24202 PMC3737333

[54] Ding W, Zhang Y, Shi S. Development and application of CRISPR/Cas in microbial biotechnology. Front Bioeng Biotechnol. 2020;8:711. 32695770 10.3389/fbioe.2020.00711 PMC7338305

[55] Mougiakos I, Mohanraju P, Bosma EF, Vrouwe V, Bou MF, Naduthodi MIS, et al. Characterizing a thermostable Cas9 for bacterial genome editing and silencing. Nat Commun. 2017;8(1):1647. 29162801 10.1038/s41467-017-01591-4 PMC5698299

[56] Couvin D, Bernheim A, Toffano-Nioche C, Touchon M, Michalik J, Néron B, et al. CRISPRCasFinder, an update of CRISRFinder, includes a portable version, enhanced performance and integrates search for Cas proteins. Nucleic Acids Res. 2018;46(W1):W246–51. 29790974 10.1093/nar/gky425 PMC6030898

[57] Aliyu H, de Maayer P, Neumann A. Not All That Glitters Is Gold: The paradox of CO-dependent hydrogenogenesis in *Parageobacillus thermoglucosidasius*. Front Microbiol. 2021;12:784652. 34956151 10.3389/fmicb.2021.784652 PMC8696081

[58] Peng C, Shi Y, Wang S, Zhang J, Wan X, Yin Y, et al. Genetic and functional characterization of multiple thermophilic organosulfur-removal systems reveals desulfurization potentials for waste residue oil cleaning. J Hazard Mater. 2023;446:130706. 36603426 10.1016/j.jhazmat.2022.130706

[59] Tourova TP, Sokolova DS, Semenova EM, Shumkova ES, Korshunova AV, Babich TL, et al. Detection of n-alkane biodegradation genes *alkB* and *ladA* in thermophilic hydrocarbon-oxidizing bacteria of the genera *Aeribacillus* and *Geobacillus*. Microbiology. 2016;85:693–707. 10.1134/S0026261716060199

[60] Sun L, Huang D, Zhu L, Zhang B, Peng C, Ma T, et al. el thermostable enzymes from *Geobacillus thermoglucosidasius* W-2 for high-efficient nitroalkane removal under aerobic and anaerobic conditions. Bioresour Technol. 2019;278:73–81. 30682639 10.1016/j.biortech.2019.01.045

[61] Cripps RE, Eley K, Leak DJ, Rudd B, Taylor M, Todd M, et al. Metabolic engineering of *Geobacillus thermoglucosidasius* for high yield ethanol production. Metab Eng. 2009;11(6):398–408. 19703579 10.1016/j.ymben.2009.08.005

[62] Lin PP, Rabe KS, Takasumi JL, Kadisch M, Arnold FH, Liao JC. Isobutanol production at elevated temperatures in thermophilic *Geobacillus thermoglucosidasius*. Metab Eng. 2014;24:1–8. 24721011 10.1016/j.ymben.2014.03.006

[63] Sheng L, Madika A, Lau MSH, Zhang Y, Minton NP. Metabolic engineering for the production of acetoin and 2,3-butanediol at elevated temperature in *Parageobacillus thermoglucosidasius* NCIMB 11955. Front Bioeng Biotechnol. 2023;11:1191079. 37200846 10.3389/fbioe.2023.1191079 PMC10185769

[64] Mohr T, Aliyu H, Küchlin R, Polliack S, Zwick M, Neumann A, et al. CO-dependent hydrogen production by the facultative anaerobe *Parageobacillus thermoglucosidasius*. Microb Cell Factories. 2018;17(1):108. 10.1186/s12934-018-0954-3 PMC6036681 29986719

[65] Imaura Y, Okamoto S, Hino T, Ogami Y, Katayama YA, Tanimura A, et al. Isolation, genomic sequence and physiological characterization of *Parageobacillus* sp. G301, an isolate capable of both hydrogenogenic and aerobic carbon monoxide oxidation. Appl Environ Microbiol. 2023;89(6):e00185-23. 37219438 10.1128/aem.00185-23 PMC10304674

[66] Kitts PA, Church DM, Thibaud-Nissen F, Choi J, Hem V, Sapojnikov V, et al. Assembly: a resource for assembled genomes at NCBI. Nucleic Acids Res. 2016;44(D1):D73-80. 26578580 10.1093/nar/gkv1226 PMC4702866

[67] Lee I, Kim YO, Park SC, Chun J. OrthoANI: An improved algorithm and software for calculating average nucleotide identity. Int J Syst Evol Microbiol. 2016;66(2):1100–3. 26585518 10.1099/ijsem.0.000760

[68] Bosi E, Donati B, Galardini M, Brunetti S, Sagot MF, Lió P, et al. MeDuSa: a multi-draft based scaffolder. Bioinformatics. 2015;31(15):2443–51. 25810435 10.1093/bioinformatics/btv171

[69] Hyatt D, Chen GL, LoCascio PF, Land ML, Larimer FW, Hauser LJ. Prodigal: prokaryotic gene recognition and translation initiation site identification. BMC Bioinformatics. 2010;11:119. 20211023 10.1186/1471-2105-11-119 PMC2848648

[70] Cantalapiedra CP, Hernández-Plaza A, Letunic I, Bork P, Huerta-Cepas J. eggNOG-mapper v2: functional annotation, orthology assignments, and domain prediction at the metagenomic scale. Mol Biol Evol. 2021;38(12):5825–9. 34597405 10.1093/molbev/msab293 PMC8662613

[71] Huerta-Cepas J, Szklarczyk D, Heller D, Hernández-Plaza A, Forslund SK, Cook H, et al. eggNOG 5.0: a hierarchical, functionally and phylogenetically annotated orthology resource based on 5090 organisms and 2502 viruses. Nucleic Acids Res. 2019;47(D1):D309-14. 30418610 10.1093/nar/gky1085 PMC6324079

[72] Yu NY, Wagner JR, Laird MR, Melli G, Rey S, Lo R, et al. PSORTb 3.0: improved protein subcellular localization prediction with refined localization subcategories and predictive capabilities for all prokaryotes. Bioinformatics. 2010;26(13):1608–15. 20472543 10.1093/bioinformatics/btq249 PMC2887053

[73] Arndt D, Grant JR, Marcu A, Sajed T, Pon A, Liang Y, et al. PHASTER: a better, faster version of the PHAST phage search tool. Nucleic Acids Res. 2016;44(W1):W16-21. 27141966 10.1093/nar/gkw387 PMC4987931

[74] Zheng J, Ge Q, Yan Y, Zhang X, Huang L, Yin Y. dbCAN3: automated carbohydrate-active enzyme and substrate annotation. Nucleic Acids Res. 2023;51(W1):W115–21. 37125649 10.1093/nar/gkad328 PMC10320055

[75] Buchfink B, Reuter K, Drost HG. Sensitive protein alignments at tree-of-life scale using DIAMOND. Nat Methods. 2021;18(4):366–8. 33828273 10.1038/s41592-021-01101-x PMC8026399

[76] Hall TA. BioEdit: a user friendly biological sequence alignment editor and analysis program for Windows 95/98/NT. Nucleic Acids Symp Ser. 1999;41(41):95–8.

[77] Emms DM, Kelly S. OrthoFinder: phylogenetic orthology inference for comparative genomics. Genome Biol. 2019;20:238. 31727128 10.1186/s13059-019-1832-y PMC6857279

[78] Wallace IM, O’Sullivan O, Higgins DG, Notredame C. M-Coffee: combining multiple sequence alignment methods with T-Coffee. Nucleic Acids Res. 2006;34(6):1692–9. 16556910 10.1093/nar/gkl091 PMC1410914

[79] Talavera G, Castresana J. Improvement of phylogenies after removing divergent and ambiguously aligned blocks from protein sequence alignments. Syst Biol. 2007;56(4):564–77. 17654362 10.1080/10635150701472164

[80] Nguyen LT, Schmidt HA, von Haeseler A, Minh BQ. IQ-TREE: a fast and effective stochastic algorithm for estimating maximum-likelihood phylogenies. Mol Biol Evol. 2015;32(1):268–74. 25371430 10.1093/molbev/msu300 PMC4271533

[81] Kalyaanamoorthy S, Minh BQ, Wong TKF, von Haeseler A, Jermiin LS. ModelFinder: fast model selection for accurate phylogenetic estimates. Nat Methods. 2017;14(6):587–9. 28481363 10.1038/nmeth.4285 PMC5453245

[82] Hoang DT, Chernomor O, von Haeseler A, Minh BQ, Vinh LS. UFBoot2: improving the ultrafast bootstrap approximation. Mol Biol Evol. 2018;35(2):518–22. 29077904 10.1093/molbev/msx281 PMC5850222

[83] Meier-Kolthoff JP, Carbasse JS, Peinado-Olarte RL, Göker M. TYGS and LPSN: a database tandem for fast and reliable genome-based classification and nomenclature of prokaryotes. Nucleic Acids Res. 2022;50(D1):D801–7. 34634793 10.1093/nar/gkab902 PMC8728197

[84] Chaudhari NM, Gupta VK, Dutta C. BPGA- an ultra-fast pan-genome analysis pipeline. Sci Rep. 2016;6(1):24373. 27071527 10.1038/srep24373 PMC4829868

[85] Zhao Y, Jia X, Yang J, Ling Y, Zhang Z, Yu J, et al. PanGP: a tool for quickly analyzing bacterial pan-genome profile. Bioinformatics. 2014;30(9):1297–9. 24420766 10.1093/bioinformatics/btu017 PMC3998138

