# Supplementary material for: Elucidating the biotechnological potential of the genera Parageobacillus and Saccharococcus through comparative genomic and pan-genome analysis
Source: BMC Genomics. 2024 Jul 25;25:723. doi: 10.1186/s12864-024-10635-1 (PMC11270796; doi:10.1186/s12864-024-10635-1)
Supplement: Supplementary file 1 — Supplementary Material 1. [file 12864_2024_10635_MOESM1_ESM.pptx]

## Slide 1
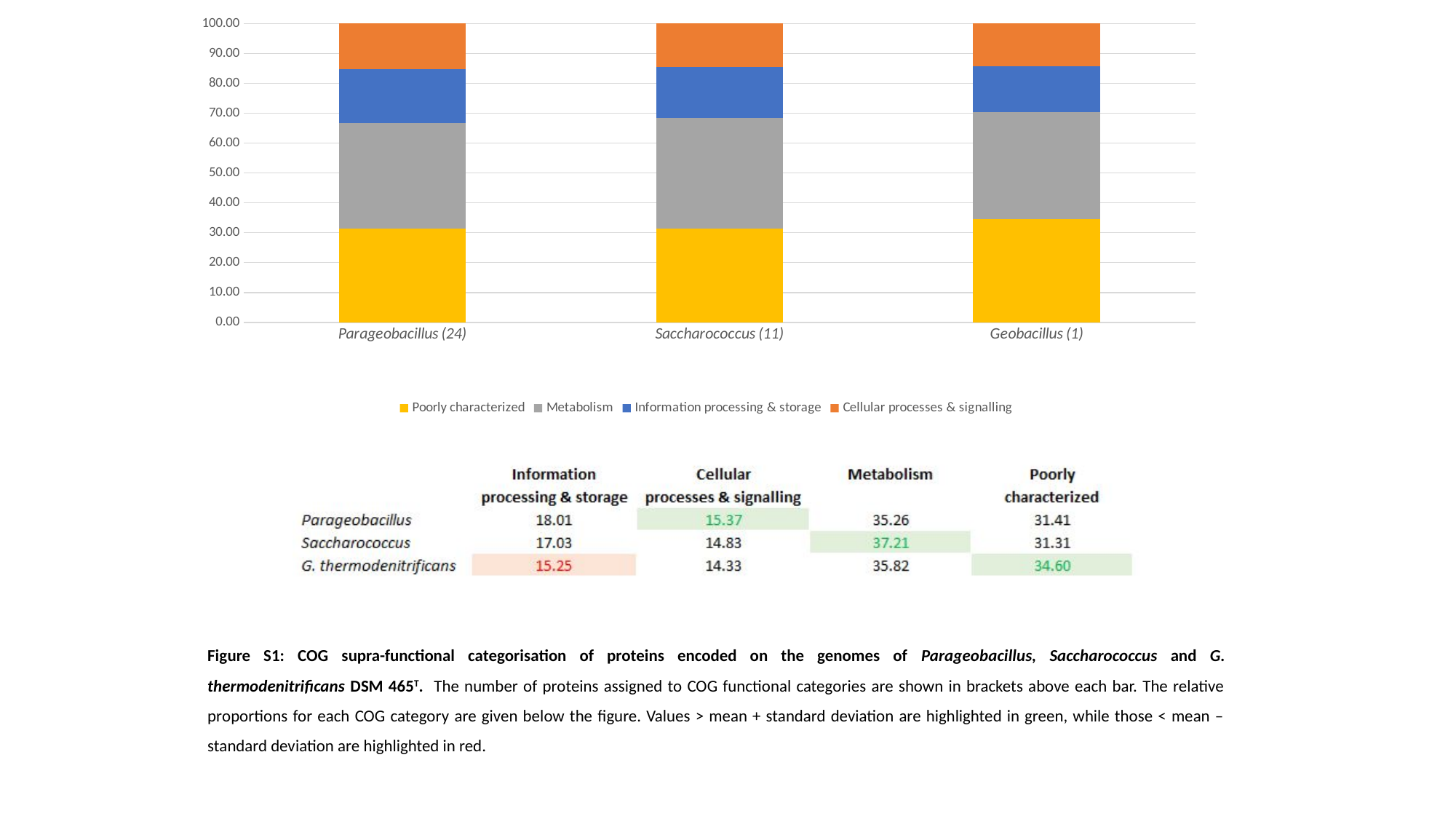

### Chart
| Category | Poorly characterized | Metabolism | Information processing & storage | Cellular processes & signalling |
|---|---|---|---|---|
| Parageobacillus (24) | 31.4136400452517 | 35.258379591553975 | 18.00546281128107 | 15.371921516979864 |
| Saccharococcus (11) | 31.30681421361443 | 37.210598959569616 | 17.031758141193396 | 14.8297216887733 |
| Geobacillus (1) | 34.60401891252955 | 35.815602836879435 | 15.24822695035461 | 14.332151300236406 |
Figure S1: COG supra-functional categorisation of proteins encoded on the genomes of Parageobacillus, Saccharococcus and G. thermodenitrificans DSM 465T. The number of proteins assigned to COG functional categories are shown in brackets above each bar. The relative proportions for each COG category are given below the figure. Values > mean + standard deviation are highlighted in green, while those < mean – standard deviation are highlighted in red.

## Slide 2
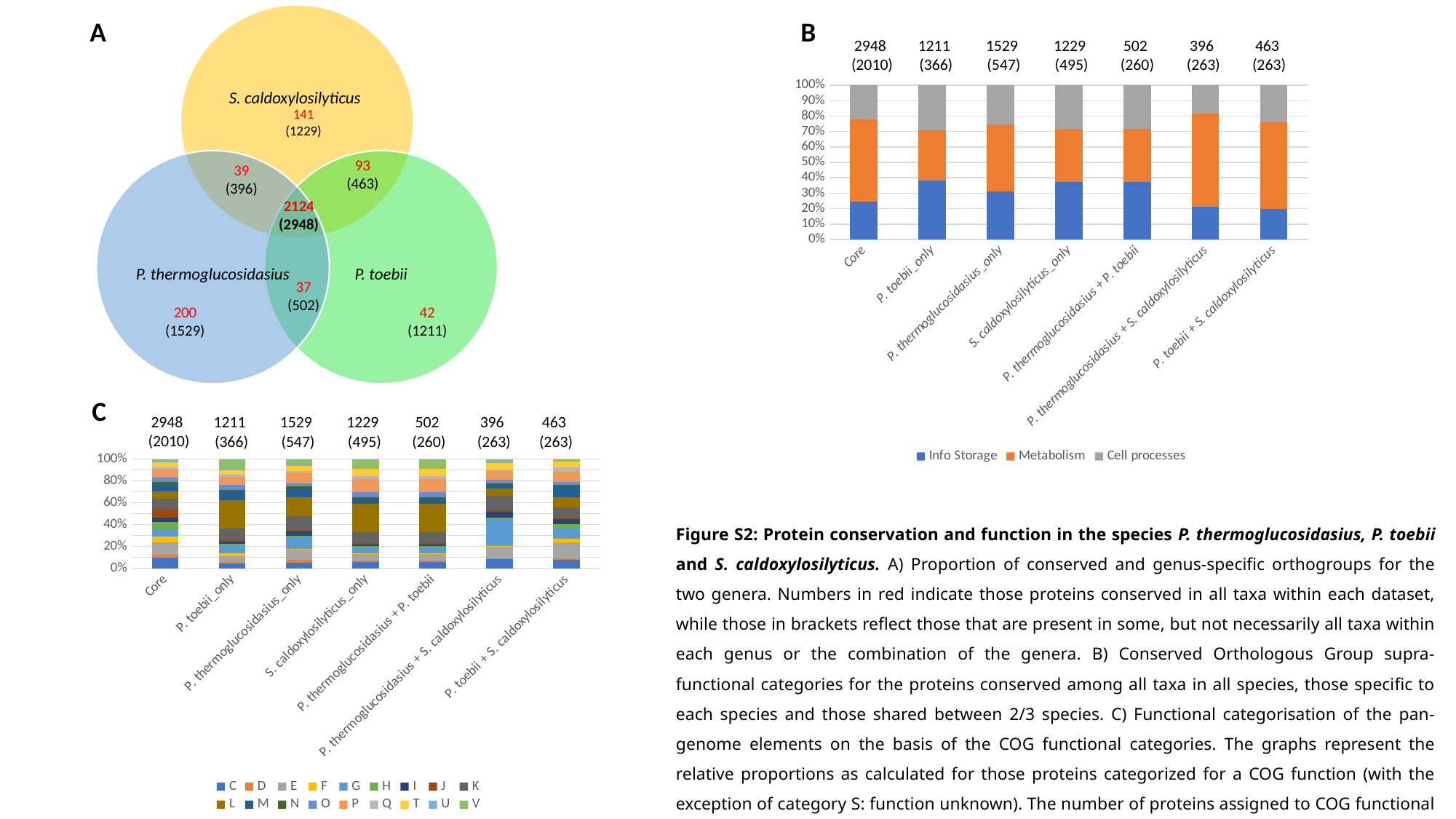

A
141
(1229)
93
(463)
39
(396)
2124
(2948)
37
(502)
200
(1529)
42
(1211)
C
B
2948
(2010)
1211
(366)
1529
(547)
1229
(495)
502
(260)
396
(263)
463
(263)
### Chart
| Category | Info Storage | Metabolism | Cell processes |
|---|---|---|---|
| Core | 24.450691585363185 | 53.26261936836994 | 22.286689046266872 |
| P. toebii_only | 38.3598939398081 | 32.391821337779845 | 29.248558073422082 |
| P. thermoglucosidasius_only | 31.350892409487315 | 43.09256392521107 | 25.55654366530161 |
| S. caldoxylosilyticus_only | 37.3079792921484 | 34.61488165293579 | 28.077139054915808 |
| P. thermoglucosidasius + P. toebii | 37.3079792921484 | 34.61488165293579 | 28.077139054915808 |
| P. thermoglucosidasius + S. caldoxylosilyticus | 21.35605593958889 | 60.1396208336185 | 18.5043232267926 |
| P. toebii + S. caldoxylosilyticus | 20.01253966142914 | 56.301559857883845 | 23.68590048068702 |2948
(2010)
1211
(366)
1529
(547)
1229
(495)
502
(260)
396
(263)
463
(263)
### Chart
| Category | C | D | E | F | G | H | I | J | K | L | M | N | O | P | Q | T | U | V |
|---|---|---|---|---|---|---|---|---|---|---|---|---|---|---|---|---|---|---|
| Core | 9.949436961362357 | 2.611727202357619 | 11.035567247249478 | 5.671179067976543 | 6.268145285658284 | 6.790490726129808 | 3.7559124529142895 | 8.506768601964815 | 8.796943930942948 | 7.146979052455421 | 5.69605266037995 | 2.628293014898287 | 3.8636648552058435 | 7.603011495579465 | 2.1888761314997183 | 4.228510708579002 | 1.7660250606418182 | 1.4924155442043534 |
| P. toebii_only | 4.236940655495722 | 1.2300795451439193 | 5.4213705819643 | 2.5968345953038297 | 6.924801137140202 | 2.1868080802558567 | 1.5034305551759015 | 1.6401060601918926 | 10.8427411639286 | 25.877046715687612 | 8.063854795943472 | 0.9567285351119373 | 4.920318180575677 | 7.198152147172183 | 2.323483585271848 | 3.8269141404477494 | 1.3667550501599104 | 8.883907826039419 |
| P. thermoglucosidasius_only | 5.398006946045548 | 2.1043077925262303 | 10.06408074686458 | 0.09149164315331436 | 10.430047319477838 | 1.6468495767596587 | 4.025632298745832 | 1.2808830041464012 | 12.656221980684283 | 17.41378742465663 | 7.95977295433835 | 1.6468495767596587 | 2.744749294599431 | 9.332147601638066 | 2.1043077925262303 | 4.696449026345934 | 1.8298328630662875 | 4.574582157665718 |
| S. caldoxylosilyticus_only | 2.929298847068378 | 0.808082440570587 | 3.063844573423381 | 0.5050515253566168 | 2.5587930480667636 | 0.808082440570587 | 0.6060618304279403 | 0.808082440570587 | 5.353546168780139 | 13.43437057448601 | 2.6262679318544078 | 0.5050515253566168 | 2.5252576267830844 | 6.4981949458483745 | 1.2121236608558805 | 3.7373812876389647 | 0.30303091521397013 | 4.242432812995581 |
| P. thermoglucosidasius + P. toebii | 5.576965976661359 | 1.5384733728720992 | 5.833121793244564 | 0.9615458580450619 | 4.871575935199502 | 1.5384733728720992 | 1.1538550296540744 | 1.5384733728720992 | 10.192386095277655 | 25.577119823998647 | 5.000038461834322 | 0.9615458580450619 | 4.80772929022531 | 12.371633627950983 | 2.3077100593081488 | 7.115439349533458 | 0.5769275148270372 | 8.07698520757852 |
| P. thermoglucosidasius + S. caldoxylosilyticus | 8.555198138388885 | 0.7604620567456787 | 10.836584308625921 | 0.570346542559259 | 23.82755762401235 | 1.711039627677777 | 5.323234397219752 | 1.330808599304938 | 13.561319857945687 | 6.463927482338269 | 4.562772340474072 | 0.38023102837283934 | 2.851732712796295 | 7.984851595829627 | 1.330808599304938 | 6.33693031886174 | 0.9505775709320983 | 2.661617198609876 |
| P. toebii + S. caldoxylosilyticus | 7.409799935401744 | 1.8999487013850624 | 14.439610130526475 | 3.6099025326316188 | 9.37282693367279 | 3.799897402770125 | 4.55987688332415 | 1.1399692208310375 | 9.436285220299052 | 9.436285220299052 | 10.639712727756349 | 0.3799897402770125 | 2.8499230520775938 | 9.499743506925313 | 3.6099025326316188 | 5.636387817528926 | 0.759979480554025 | 1.51995896110805 |Figure S2: Protein conservation and function in the species P. thermoglucosidasius, P. toebii and S. caldoxylosilyticus. A) Proportion of conserved and genus-specific orthogroups for the two genera. Numbers in red indicate those proteins conserved in all taxa within each dataset, while those in brackets reflect those that are present in some, but not necessarily all taxa within each genus or the combination of the genera. B) Conserved Orthologous Group supra-functional categories for the proteins conserved among all taxa in all species, those specific to each species and those shared between 2/3 species. C) Functional categorisation of the pan-genome elements on the basis of the COG functional categories. The graphs represent the relative proportions as calculated for those proteins categorized for a COG function (with the exception of category S: function unknown). The number of proteins assigned to COG functional categories are shown in brackets above each bar.
